# Biomimetic Co‐delivery of Lenvatinib and FePt Nanoparticles for Enhanced Ferroptosis/Apoptosis Treatment of Hepatocellular Carcinoma

**DOI:** 10.1002/adhm.202401747

**Published:** 2025-03-21

**Authors:** Feichao Xuan, Xingyang Zhao, Weiran Pang, Zirong Li, Xiangyi Yin, Weizhong Xie, Xiaojun Zeng, Liming Nie, Junying Yang, Shiying Li, Puxiang Lai, Chihua Fang

**Affiliations:** ^1^ Department of Hepatobiliary Surgery I Zhujiang Hospital Southern Medical University Guangzhou 510280 China; ^2^ Department of Biomedical Engineering The Hong Kong Polytechnic University Hong Kong 999077 China; ^3^ Medical Research Institute Guangdong Provincial People's Hospital (Guangdong Academy of Medical Sciences) Southern Medical University Guangzhou 510080 China

**Keywords:** apoptosis, cancer cell membrane coated nanoparticles, combined therapy, ferroptosis, hepatocellular carcinoma, Lenvatinib

## Abstract

Lenvatinib, endorse as a first‐line targeted therapy, has demonstrated efficacy in extending the survival span of individuals afflicted with advanced Hepatocellular carcinoma (HCC). However, its therapeutic effect wears off with time, which is ascribed to the cancer cell's tendency to evade and tamper with its usual modes of action, severely limiting its clinical use. This study devises an innovative therapeutic modality involving the synergistic co‐delivery of FePt nanoparticles (NPs) and Lenvatinib via poly lactic‐co‐glycolic acid (PLGA) NPs encase in HCC cell membranes (Len/FePt@CMP NPs). The investigation explores the mechanism through which Lenvatinib induces ferroptosis in HCC, notably by dampening the glutathione peroxidase 4 (GPX4) through the inhibition of fibroblast growth factor receptor 4. FePt NPs are engineered to enhance the efficacy of ferroptosis and apoptosis for HCC treatment. Concurrently, the incorporation of the cancer cell membrane facilitates the targeted accumulation of NPs at the tumor site, leveraging mechanisms of immune evasion and homologous targeting. This enhances ferroptosis/apoptosis treatment efficacy, triggeres by Len/FePt@CMP NPs, is convincingly demonstrated both in vitro and in vivo. The proposed approach has the potential to redefine HCC therapeutic paradigms by overcoming mono‐therapeutic limitations in current clinical treatments, showcasing the improved efficacy of a comprehensive strategy.

## Graphical abstract

This study introduces a novel HCC treatment using poly lactic‐co‐glycolic acid (PLGA) nanoparticles encased in HCC cell membranes to co‐deliver Lenvatinib and FePt nanoparticles. This approach improves treatment efficacy by enhancing ferroptosis and apoptosis. Significant results are demonstrated both in vitro and in vivo, suggesting a promising new therapeutic strategy for advanced HCC in clinical setting.

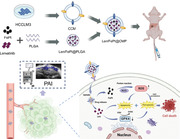

## 1 Introduction

Hepatocellular carcinoma (HCC) is the fourth primary cause of cancer mortality globally, exhibiting a relatively low five‐year survival rate of merely 18%, which constitutes a severe challenge to public health.^[^ ^1^ ^]^ In a substantial majority of HCC patients, the disease onset is insidious, resulting in a situation where, by the time symptoms become evident and a diagnosis is made, the condition has often progressed to an advanced stage. For tumors that are not amenable to surgical resection or are poorly specific for chemotherapy, targeted molecular agents have in recent years provided new horizons for extending the survival of HCC patients.^[^ ^2^ ^]^ Lenvatinib (Len) is a multi‐targeted tyrosine kinase inhibitor that was authorized as the second first‐line medication for the molecular targeting of HCC after sorafenib in 2018. It suppresses vascular endothelial growth factor receptor 1–3, fibroblast growth factor receptor (FGFR) 1–4, platelet‐derived growth factor receptor, as well as rearranged proto‐oncogene during transfection to achieve anti‐angiogenesis and tumor growth inhibition, which improves the prognosis of patients.^[^ ^3^ ^]^ Additionally, Len is known to modulate the apoptotic pathway in cancer cells through the downregulation of B‐cell lymphoma‐2 and Myeloid cell leukemia 1 proteins and the activation of caspase enzymes.^[^ ^4^ ^]^ However, these antitumor effects are often restricted by various factors, such as alterations in signaling pathways, dysregulation of apoptosis, modulations in the tumor microenvironment, involvement of cancer stem cells, changes in drug metabolism/transport, and DNA repair, etc.^[^ ^5^ ^]^ Currently, patients exhibit an overall response rate of 24.1% to Len treatment, demonstrating reduced responsiveness during therapy, which significantly limits its clinical efficacy.^[^ ^6^ ^]^ Moreover, poor solubility of Len in bodily fluids and blood compromises its bioavailability and distribution, hindering its accumulation at the tumor site and necessitating higher dosages for therapeutic efficacy. These increased dosages, however, are associated with a spectrum of adverse effects, such as hypertension, diarrhea, anorexia, weight loss, fatigue, palmar‐plantar erythrodysesthesia syndrome, proteinuria, and others.^[^ ^7^ ^]^ Consequently, there exists a pressing need to elucidate the underlying molecular mechanisms and exploit innovative therapeutic strategies that can augment the therapeutic efficacy of Len in HCC management.

Ferroptosis, delineated as an iron‐dependent process characterized by the accumulation of reactive oxygen species (ROS) and cytotoxic lipid peroxides (LPO),^[^ ^8^ ^]^ represents a novel paradigm of programmed cell death.^[^ ^9^ ^]^ This form of cell death plays a pivotal role in numerous physiological functions and a spectrum of diseases, notably cancer.^[^ ^10^ ^]^ Emerging research underscores the potential of ferroptosis induction as a therapeutic strategy in oncology.^[^ ^11^ ^]^ Recent progress has elucidated that Len promotes ferroptosis through the inhibition of FGFR4, suppression of the system Xc^−^ (xCT) and reduction of glutathione peroxidase 4 (GPX4) expression.^[^ ^12^ ^]^ The xCT transporter, an essential component of the cellular antioxidant defense system, is ubiquitously expressed across the phospholipid bilayer. Cystine and glutamate undergo cellular exchange through the xCT in a 1:1 ratio, where absorbed cystine is reduced to cysteine within the cell and participates in the synthesis of glutathione (GSH).^[^ ^13^ ^]^ GPX4 plays a pivotal role in this antioxidant pathway by catalyzing the conversion of GSH into its oxidized form and reducing cytotoxic lipid peroxides to non‐toxic lipid alcohols,^[^ ^14^ ^]^ thereby preventing the accumulation of LPO. The xCT‐GSH‐GPX4 pathway is recognized as a critical inhibitor of ferroptosis, embodying a significant component of the organism's defense against oxidative stress and LPO‐mediated cellular damage. As a result, amplifying the ferroptosis induced by Len could serve as a potential mechanism to augment therapeutic outcomes. However, investigations into Len‐induced ferroptosis remain limited.

As an efficient ferroptosis reagent, FePt nanoparticles (NPs) could release active Fe^2+^ in the acidic tumor microenvironment and catalyze the Fenton reaction to generate the highly toxic hydroxyl radical, a form of ROS that causes phospholipid peroxidation.^[^ ^15^ ^]^ Simultaneously, hydroxyl radical can also oxidize organic macromolecules within cancer cells, leading to apoptosis.^[^ ^16^ ^]^ Consequently, the concomitant use of FePt NPs with Len could potentially amplify the efficacy of both ferroptosis and apoptosis. Owing to their platinum content, FePt NPs exhibit exceptional contrast‐enhancing properties for photoacoustic imaging (PAI).^[^ ^16a^ ^]^ As an innovative noninvasive imaging modality, PAI transcends the constraints of traditional optical and ultrasound imaging by providing robust optical contrast and relatively high spatial resolution in deep tissue environments.^[^ ^17^ ^]^ Accordingly, PAI has been increasingly utilized across a spectrum of biological research domains, including early tumor diagnosis, metabolic monitoring, and nanomaterials in‐vivo tracking, heralding new frontiers in medical diagnosis and treatment monitoring.^[^ ^18^ ^]^

With the development of nanotechnology, nanocarrier delivery has brought numerous advantages to cancer drug therapy, such as protecting the drug from biodegradation, controlling drug distribution in vivo, reducing systemic toxicity, and enhancing therapeutic efficacy.^[^ ^19^ ^]^ Poly lactic‐co‐glycolic acid (PLGA) is a biodegradable functional polymer organic compound known for its excellent biocompatibility, spheronization, and non‐toxic properties, which has been widely used in drug delivery and other medical applications in recent years. Despite their advantages, NPs still encounter challenges such as biological instability, inadequate targeting, and expedited clearance by the reticuloendothelial system (RES) in biological application.^[^ ^20^ ^]^ The innovation of cell membrane biomimetic technology emerges as a strategic solution to these obstacles.^[^ ^21^ ^]^ Utilizing different cell membrane coatings endows NPs with specific functionalities. For instance, erythrocyte membrane‐coated NPs can circumvent immune surveillance,^[^ ^22^ ^]^ whereas those cloaked with stem cell membranes exhibit potent anti‐tumor targeting properties.^[^ ^23^ ^]^ Remarkably, encapsulating NPs within cancer cell membranes is a proficient approach for drug delivery, leveraging immune evasion and homologous targeting capabilities intrinsic to the membrane proteins of cancer cells.^[^ ^24^ ^]^ The majority of advanced NPs exploit the enhanced permeability and retention (EPR) effect, resultant from the aberrant tumor vasculature, to achieve passive tumor targeting. Beyond EPR‐mediated targeting, cancer cell membrane‐coated NPs can actively accumulate at tumor sites via homologous targeting mechanisms. Research conducted by Fang et al. demonstrated that the tumor cell uptake efficiency of cancer cell membrane‐coated NPs could be 40‐fold higher compared to uncoated NPs.^[^ ^25^ ^]^ Additionally, the immune evasion property of the cancer cell membrane can facilitate evasion of RES‐mediated phagocytosis, thereby extending the circulatory lifespan of the NPs.

**Scheme 1:**
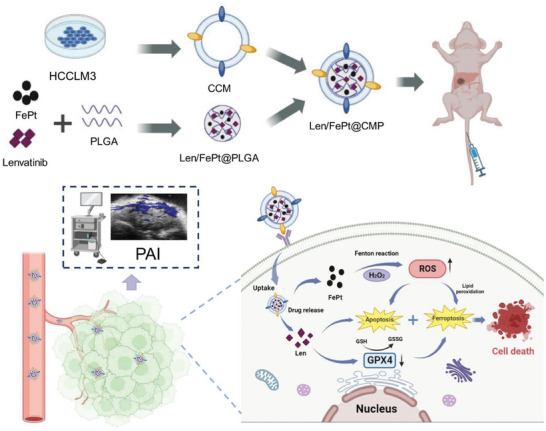
Synthesis of Len/FePt@CMP NPs for targeted and enhanced apoptosis/ferroptosis synergetic therapy in HCC.

Herein, we propose a novel therapeutic strategy that involves the use of HCC cell (HCCLM3) membrane‐coated PLGA NPs to encapsulate Len and FePt NPs (Len/FePt@CMP NPs) for targeted and enhanced ferroptosis/apoptosis therapy in HCC (**Scheme** **1**). The application of HCC cell membrane coating is designed to prolong the circulation time of the NPs in the bloodstream, improve their biocompatibility, and facilitate active targeting to tumor sites. Due to the enrichment of Len/FePt@CMP NPs at the tumor site, the dosage and side effects of the drugs can be significantly reduced, thereby alleviating patient suffering during chemotherapy. Furthermore, accelerating the Fe^2+^‐catalyzed lipid peroxidation by FePt NPs, combined with the inhibition of the xCT‐GSH‐GPX4 pathway by Len, is proposed to synergistically enhance the effectiveness of ferroptosis therapy. Specifically, FePt NPs are posited to promote ferroptosis through positive regulatory mechanisms, whereas Len is suggested to counteract the body's innate negative regulators of ferroptosis. Besides, the pro‐apoptotic effects of both provide extra therapeutic benefits. Additionally, the accumulation and metabolism of Len/FePt@CMP NPs can be tracked by PAI. The enhanced ferroptosis/apoptosis of Len/FePt@CMP NPs leads to remarkable therapeutic effects for HCC, surpassing any single therapy strategy. Moreover, this research contributes to provide further evidence supporting the role of Len in inducing ferroptosis, highlighting its potential utility in the context of cancer therapy.

## 2 Results and Discussion

### 2.1 Characterization of Len/FePt@CMP NPs

**Figure 1:**
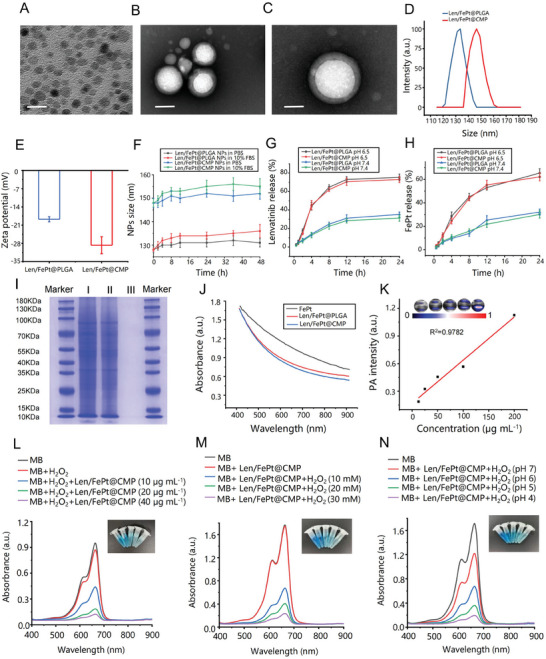
Characterization of the synthesized Len/FePt@CMP NPs. A) TEM images of FePt NPs (scale bar: 20 nm). B) TEM images of Len/FePt@CMP NPs (scale bar: 100 nm). C) TEM images of Len/FePt@CMP NPs (scale bar: 50 nm). D) Hydrated particle size of Len/FePt@CMP and Len/FePt@PLGA NPs. E) The surface potentials of Len/FePt@CMP and Len/FePt@PLGA NPs. F) Time‐dependent stability of Len/FePt@CMP and Len/FePt@PLGA NPs in 10% fetal bovine serum and phosphate‐buffered saline solution at 37^ °^C. G) The Len release curve of Len/FePt@CMP and Len/FePt@PLGA NPs at pH 6.5 and 7.4 using HPLC. H) The FePt NPs release curve of Len/FePt@CMP and Len/FePt@PLGA NPs at pH 6.5 and 7.4 using ICP‐OES. I) Sodium dodecyl sulfate‐polyacrylamide gel electrophoresis protein marker analysis of I (HCCLM3 cell membranes), II (Len/FePt@PLGA NPs), and III (Len/FePt@PLGA NPs). J) The UV absorption spectrum of FePt, Len/FePt@PLGA, and Len/FePt@CMP NPs. K) PA images and signal of Len/FePt@CMP NPs. L) MB absorbance after incubation with various concentrations of Len/FePt@CMP NPs and H_2_O_2_ (30 mm) at pH 5 value. M) MB absorbance after incubation with Len/FePt@CMP NPs (40 µg mL^−1^) and various concentrations of H_2_O_2_ at pH 5 value. N) MB absorbance after incubation with Len/FePt@CMP NPs (40 µg mL^−1^) and H_2_O_2_ (30 mm) at various pH values.

FePt NPs were synthesized via a thermo‐reduction method, and Len/FePt@CMP NPs were prepared following established protocols.^[^ ^16^ ^]^ Transmission electron microscopy (TEM) analysis revealed that both FePt NPs and Len/FePt@CMP NPs possessed uniform spherical morphologies with diameters of ≈7  and 140 nm, respectively, as depicted in **Figure** **1**A,B. Notably, a cellular membrane was distinctly showed at the Len/FePt@CMP NPs surface (Figure 1C). Dynamic light scattering (DLS) measurements indicated the hydrodynamic diameters of Len/FePt@PLGA NPs and Len/FePt@CMP NPs to be 128.29±12.48 nm and 148.91±16.23 nm, respectively (Figure 1D). Zeta potential analysis demonstrated that the membrane‐coated NPs had a surface charge of −28.72 mV, in contrast to −18.74 mV for Len/FePt@PLGA NPs (Figure 1E), which contributes to preventing nanoparticle aggregation and adsorption, thereby ensuring stable dispersion. Encapsulation efficiency and drug loading efficiency for Len within Len/FePt@CMP NPs were determined to be 78.8% and 5.13%, respectively, as assessed by high‐performance liquid chromatography (HPLC) (Figure S1A, Supporting Information). Similarly, FePt NPs demonstrated encapsulation and drug loading efficiencies of 60.42% and 3.26%, respectively, measured via inductively coupled plasma pptical emission spectrometry (ICP‐OES) (Figure S1B, Supporting Information). Encapsulation efficiency and drug loading efficiency for Len within Len@CMP NPs were detected to be 80.8% and 5.25%; FePt NPs demonstrated encapsulation and drug loading efficiencies of 63.2% and 3.42%. The Len/FePt@CMP and Len/FePt@PLGA NPs have good colloidal stability in PBS or 10% FBS (Figure 1F). Drug release profiles indicated a gradual release of Len and FePt NPs from both Len/FePt@PLGA NPs and Len/FePt@CMP NPs, suggesting that the membrane coating does not impede drug release (Figure 1G,H). At pH 6.5 conditions, mimicking the tumor microenvironment, the drug release rate of the nanoparticles was faster because the PLGA core decomposed more easily in acidic conditions. Further, the proteins of HCCLM3 cell membranes, Len/FePt@CMP NPs, and Len/FePt@PLGA NPs were analyzed via sodium dodecyl sulfate–polyacrylamide gel electrophoresis (SDS‐PAGE). The proteins were retained on the cell membranes and in Len/FePt@CMP NPs, even after hypotonic treatment and extrusion. As shown in Figure 1I, the cell membrane proteins of the HCCLM3 cell membranes were successfully transferred to Len/FePt@CMP NPs. Additionally, western blot (WB) analysis confirmed the presence of CD47 on Len/FePt@CMP NPs (Figure S2A,B, Supporting Information), a “don't eat me” signaling molecule that was highly expressed on the HCC cell membrane, which enabled the escape of them from macrophages.^[^ ^26^ ^]^ The above results all indicated successful synthesis of drug‐loaded nanoparticles, which have been encapsulated by the HCC cell membrane. Moreover, the UV absorption spectrum of Len/FePt@CMP NPs exhibited strong absorption within the wavelength range of 400–900 nm, even though no specific absorbance peak is detected, which preliminarily demonstrates that the NPs are competent for high‐performance photoacoustic imaging (Figure 1J). Photoacoustic (PA) signal intensity of Len/FePt@CMP NPs correlated positively (R^2^ = 0.9782) with probe concentration, highlighting their potential for PA imaging applications (Figure 1K). Finally, the generation of hydroxyl radicals (·OH) by Len/FePt@CMP NPs in a simulated microenvironment was evaluated using Methylene Blue (MB), a dye sensitive to ·OH‐mediated decolorization. Significant reductions in MB absorbance were observed when the dye was incubated with Len/FePt@CMP NPs and H_2_O_2_ under acidic conditions, demonstrating the nanoparticles' capacity for ·OH generation in tumor microenvironment (Figure 1L–N).

### 2.2 Specificity Evaluation of HCC Cell Membrane Coated NPs

**Figure 2:**
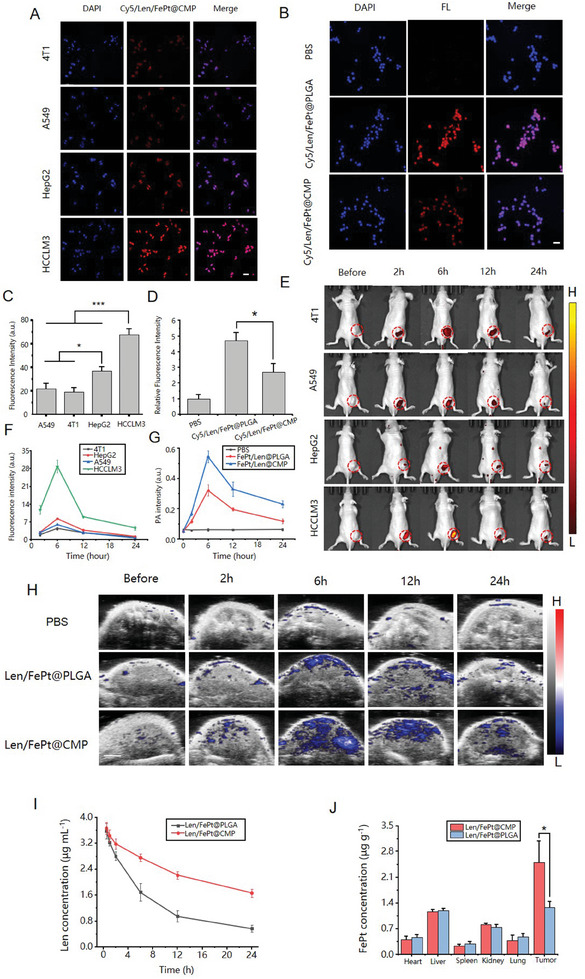
In‐vitro/vivo evaluation of immune escape and homotypic targeting capabilities of Len/FePt@CMP NPs. A) Fluorescence images of HCCLM3, A549, HepG2, and 4T1 cells treated with Cy5/Len/FePt@CMP NPs (scale bar: 50 µm). B) Fluorescence images of RAW264.7 cells treated with Cy5/Len/FePt@CMP NPs, Cy5/Len/FePt@PLGA NPs, and PBS (scale bar: 50 µm). C) Quantification of fluorescence intensity in A (^*^ *p*‐value < 0.05). D) Quantification of fluorescence intensity in B (^***^ *p*‐value < 0.001). E) Real‐time fluorescence images of the HCCLM3, HepG2, A549, and 4T1 tumor‐bearing mice after injection of Cy5/Len/FePt@CMP NPs. Red circles show the tumor regions. F) Quantitative analysis of fluorescence intensity analysis at the tumor site at different time points (*n* = 3). G) Quantitative analysis of PA signals at the tumor site (*n* = 3). H) In vivo PA imaging of HCCLM3 tumor‐bearing mice after injection with Len/FePt@CMP NPs, Len/FePt@PLGA NPs and PBS (scale bar: 1 mm). I) In vivo blood circulation by quantifying Len concentration at different time points after intravenous injection of Len/FePt@CMP and Len/FePt@PLGA NPs using HPLC. J) Evaluation of Len/FePt@CMP NPs distribution in various organs by FePt NPs detection using ICP‐OES (^*^ *p*‐value < 0.05).

The Len/FePt@CMP NPs were coated with a HCC cell membrane, hypothesized to confer immune evasion and homologous targeting functionalities. To verify these attributes, Cy5 dye was encapsulated within the core of Len/FePt@CMP NPs for tracking purposes. Incubation of Cy5/Len/FePt@CMP NPs with a panel of tumor cells, including HCCLM3 (hepatocellular carcinoma cells) and HepG2 (liver tumor cells), A549 (lung cancer cells), and 4T1 (breast cancer cells) for 4 h revealed the highest uptake of HCCLM3 cells, as demonstrated by the most pronounced fluorescence signal compared with the others (**Figure** **2**A, C). This observation implied a targeted interaction mediated by homologous targeting moieties present on the nanoparticle surface, facilitating specific recognition and binding to HCCLM3 cells. Furthermore, intracellular Fe^2+^ released by Len/FePt@CMP NPs was quantitatively analyzed using the FerroOrange fluorescence staining method, in which the fluorescence intensity was enhanced due to the enrichment of Fe^2+^ in the cells. HCCLM3 cells treated with Len/FePt@CMP NPs demonstrated significantly higher fluorescence intensity compared to other cells, indirectly substantiating the homologous targeting capability of designed NPs (Figure S3A,B, Supporting Information). Notably, HepG2 cells also showed elevated fluorescence intensity relative to A549 and 4T1 cells in both experiments, suggesting a degree of targeting affinity of Len/FePt@CMP NPs towards other liver tumor types, potentially due to shared liver tumor‐specific antigens. The antiphagocytic property of Len/FePt@CMP NPs was assessed through its uptake by RAW264.7 macrophages, as verified by fluorescence microscopy. Following a 4 h co‐incubation with RAW264.7 macrophages, the Cy5/Len/FePt@CMP NPs treated group exhibited a diminished fluorescence signal relative to the Cy5/Len/FePt@PLGA control group (Figure 2B,D), suggesting a reduced phagocytic uptake attributed to enhanced immune evasion capabilities.These findings collectively indicate that in vitro Len/FePt@CMP NPs not only effectively avoids immune engulfment but also targets HCCLM3 tumors, demonstrating excellent tumor specificity in vitro.

To evaluate the in vivo homologous targeting capability of Len/FePt@CMP NPs, subcutaneous tumor mouse models were established with four type tumor cells, mirroring the preceding in vitro analyses. Cy5/Len/FePt@CMP NPs were administered intravenously via the tail vein and the tumor site accumulations of NPs were monitored by the IVIS Imaging Spectrum System. Results indicated that the accumulations increased and peaked at 6 h post‐injection, then subsequently diminished over time (Figure 2E). Notably, fluorescence signals from HCCLM3 tumors consistently outperformed those from other tumor types at each time point, suggesting effective in vivo targeting of homologous HCCLM3 tumors by Len/FePt@CMP NPs (Figure 2F). To further demonstrate this active targeting effect, we employed PA imaging to investigate the accumulation and deep‐seated information of Len/FePt@CMP NPs in HCCLM3 tumors. Similarly, the maximum tumor accumulation was observed at 6 h after injection (Figure 2H). Mice injected with Len/FePt@CMP NPs consistently exhibited stronger PA signals at all time points compared to those injected with Len/FePt@PLGA NPs and it also demonstrated the ability of Len/FePt@CMP NPs to penetrate deeper into the tumor (Figure 2G). Notably, high PA signals remained at 12 h post‐injection, suggesting that Len/FePt@CMP NPs can retain in the vicinity of the tumor for long periods of time, highlighting its potential in guiding cancer treatment and diagnostic processes in vivo. Furthermore, Len concentration in the mice's bloodstream was quantified at various time points using HPLC, revealing that the Len/FePt@CMP group maintained higher Len levels than the Len/FePt@PLGA group, implying that the cell membrane coating extends the nanoparticles' circulatory longevity because of its antiphagocytic effects against the reticuloendothelial system (Figure 2I). After PA imaging, tumors and vital organs were excised for additional comparative analysis of Len/FePt@CMP NPs and Len/FePt@PLGA NPs distribution via ICP‐OES. As illustrated in Figure 2J, cancer cell membrane coating markedly enhanced drug accumulation at the tumor site due to its active homologous targeting effect. This result also showed the distribution of Len/FePt@CMP NPs in major organs. Collectively, these findings substantiate the homologous targeting and immune evasion capabilities Len/FePt@CMP NPs in vivo, reinforcing their therapeutic potential.

### 2.3 Therapeutic Efficacy of Len/FePt@CMP NPs in HCC Cells

**Figure 3:**
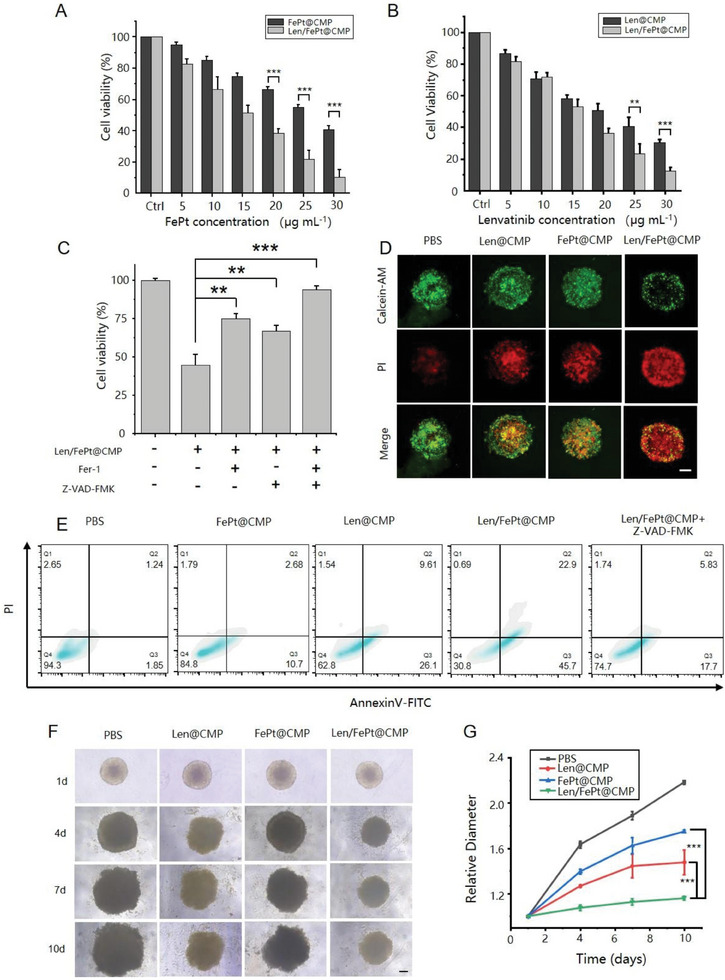
Therapeutic efficacy of Len/FePt@CMP NPs for HCCLM3 cells. A) The cell viability of HCCLM3 cells after treatment with different concentrations of Len/FePt@CMP and FePt@CMP NPs (^***^ *p*‐value < 0.001). B) The cell viability of HCCLM3 cells after treatment with different concentrations of Len/FePt@CMP and Len@CMP NPs (^***^ *p*‐value < 0.001). C) The cell viability of HCCLM3 cells after various treatments (^***^ *p*‐value < 0.001 and ^**^ *p*‐value < 0.01). D) Calcein‐AM/PI co‐staining of HCCLM3 cell sphere model after various treatments (green: live cells; red: dead cells; scale bar: 150 µm). E) Flow cytometry analyses of cell apoptosis by Annexin V‐FITC/PI co‐staining after different treatments. F) Images of tumor spheroids after various treatments in ten days (scale bar: 150 µm). G) Diameter changes of tumor spheroids in different treated groups (^***^ *p*‐value < 0.001).

As Len/FePt@CMP NPs can be effectively attracted by homologous cancer cells, we subsequently validated the therapeutic efficacy of Len/FePt@CMP NPs in a series of in vitro experiments. First, the cytotoxic effects of NPs on HCCLM3 cells were measured using the CCK‐8 assay. Results indicated that the IC50 values (the drug concentrations required to induce 50% cell death within a certain period) for FePt@CMP and Len@CMP NPs were 28.23  and 21.12 µg mL^−1^, respectively, after a 24 h incubation with HCCLM3 cells. In contrast, Len/FePt@CMP NPs demonstrated reduced IC50 values of 13.12 µg mL^−1^ in **Figure** **3**A and 16.73 µg mL^−1^ in Figure 3B, thereby showcasing superior cytotoxicity relative to the other two NPs. However, when Len/FePt@CMP NPs were combined with Ferrostatin‐1(Fer‐1) or benzyloxycarbonyl‐Val‐Ala‐Asp‐fluoromethylketone (Z‐VAD‐FMK), the cell survival rate significantly increased (Figure 3C), indicating the critical role of NPs‐induced ferroptosis and apoptosis in killing HCC cells. Flow cytometry analysis, following annexin V‐FITC/PI staining, elucidated the cell death pathway induced by Len/FePt@CMP NPs in comparison to the control groups. Apoptotic rates were ≈5.7%, 15.2%, 37.2%, 69.2%, and 25.3% for the PBS, FePt@CMP, Len@CMP, Len/FePt@CMP, and Len/FePt@CMP+Z‐VAD‐FMK groups, respectively, similar to the cytotoxicity trends observed in the CCK8 assay and underscoring the therapeutic potency of Len/FePt@CMP NPs (Figure 3E). Additionally, to further simulate tumor behavior and physiological conditions, a 3D cell sphere model was employed for the evaluation of Len/FePt@CMP effects. The cell spheres were treated with PBS, FePt@CMP, Len@CMP, and Len/FePt@CMP NPs respectively, when they reached a diameter of approximately 600 µm in the spherical micro‐well plate. The anti‐tumor efficacy of Len/FePt@CMP NPs was further substantiated via calcein‐AM (denoting live cells with green fluorescence) and propidium iodide (PI, indicating dead cells with red fluorescence) staining. Fluorescence microscopy images underscored the pronounced anti‐tumor activity of Len/FePt@CMP NPs. In contrast, neither FePt@CMP nor Len@CMP NPs treatment alone was able to effectively kill the cancer cells (Figure 3D). Continuous monitoring of spheroid growth and diameter measurement revealed minimal size alterations in the Len/FePt@CMP‐treated group, whereas significant growth and expansion were noted in the other groups (Figure 3F,G). This observation corroborates the exceptional inhibitory effect of Len/FePt@CMP NPs on tumor proliferation. Based on these positive results, the combined strategy of Len/FePt@CMP NPs demonstrated significant advantages in killing HCC cells.

### 2.4 Validation of Enhanced Apoptosis and Ferroptosis in HCC Cells

**Figure 4:**
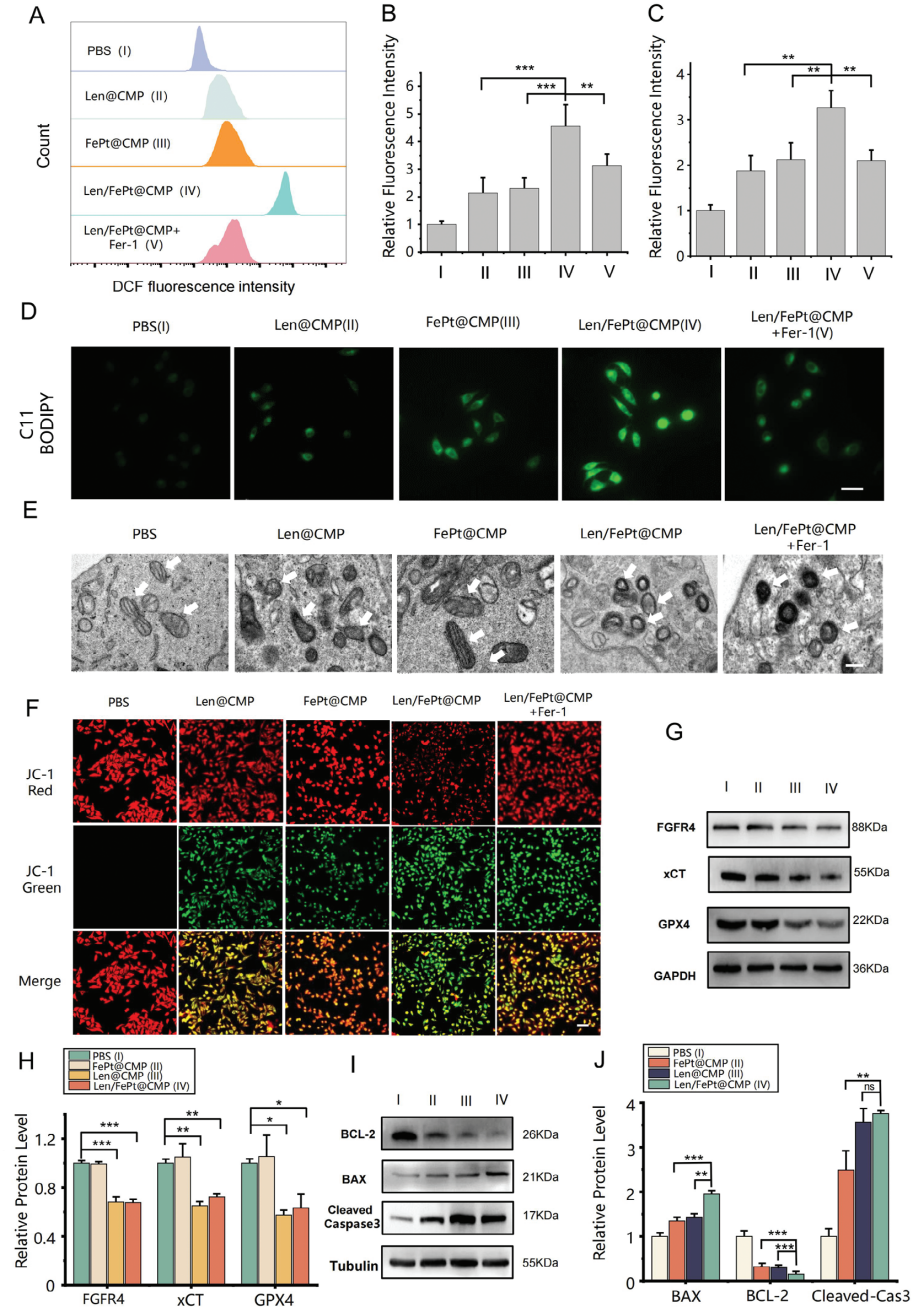
Enhanced ferroptosis/apoptosis effect validation in vitro. A) ROS detected by DCFH‐DA in HCCLM3 cells. B) Quantification of DCF fluorescence intensity analysis in A (^***^ *p*‐value < 0.001). C) Quantification of fluorescence intensity analysis in D (^**^ *p*‐value < 0.01). D) LPO detected by C11 BODIPY in HCCLM3 cells (scale bar: 50 µm). E) Mitochondria performance showing with TEM. The white arrows indicate the positions of mitochondria (scale bar: 1 µm). F) Mitochondrial membrane potential detected by JC‐1 probe in HCCLM3 cells (scale bar: 50 µm). G) WB analysis on the expression of key ferroptosis markers in HCCLM3 cells after treatments. H) Quantitative measurement of ferroptosis‐associated proteins in G (^*^ *p*‐value < 0.05, ^**^ *p*‐value < 0.01, and ^***^ *p*‐value < 0.001). I) WB analysis on the expression of key apoptosis markers in in HCCLM3 cells after treatments. J) Quantitative measurement of apoptosis‐associated proteins in I (^**^ *p*‐value < 0.01, ^***^ *p*‐value < 0.001).

After revealing the therapeutic effects of Len/FePt@CMP NPs in vitro, we proceeded to validate intracellular enhancement of apoptosis/ferroptosis effects and mechanisms facilitated by this compound. Within the tumor microenvironment, Len/FePt@CMP NPs disintegration leaded to Fe^2+^ release from FePt NPs, then ROS is generated under the Fenton reaction. Concurrently, Len targeted the xCT‐GSH‐GPX4 signaling pathway, curtailing GSH production and diminishing GPX4 expression. The release of Fe^2+^ was verified via FerroOrange fluorescence staining, which demonstrated increased fluorescence intensity proportional to intracellular Fe^2+^ accumulation (Figure S4A, Supporting Information). HCCLM3 cells treated with Len/FePt@CMP NPs exhibited a significantly enhanced fluorescence signal compared to those treated with FePt@PLGA NPs and PBS (Figure S4B, Supporting Information), further indicating that NPs coated with cancer cell membranes facilitated superior cellular uptake. Cellular ROS levels were quantified using 2′,7′‐dichlorodihydrofluorescein diacetate (DCFH‐DA), a probe oxidized by ROS to 2′,7′‐dichlorofluorescein (DCF). Flow cytometry used to quantify DCF fluorescence intensity showed that HCCLM3 cells exposed to Len/FePt@CMP NPs exhibited the strongest fluorescence intensity, indicating elevated ROS levels compared to the other control groups. (**Figure** **4**A,B). This oxidative stress, attributed to increased ROS and reduced GPX4, potentially leads to LPO accumulation, which could be assessed via the C11 BODIPY probe. The Len/FePt@CMP NPs treatment group exhibited the highest LPO production, indicating a potentiated ferroptosis effect (Figure 4C,D). Furthermore, alterations in tumor mitochondria are another crucial ferroptosis‐associated feature. TEM images of cells treated by Len/FePt@CMP NPs showed a noticeable reduction in mitochondrial size, an increase in mitochondrial membrane density, and the disappearance of mitochondrial cristae, all of which are typical mitochondrial morphological characteristics of ferroptosis (Figure 4E). In addition, ferroptosis results in a decrease or disappearance of mitochondrial membrane potential, which can be detected using the JC‐1 probe. High mitochondrial membrane potential results in JC‐1 forming J‐aggregates that emit red fluorescence, whereas low potential causes JC‐1 to remain as monomers emitting green fluorescence. The Len/FePt@CMP group exhibited pronounced green fluorescence, indicating significant mitochondrial membrane depolarization (Figure 4F).

Western blot (WB) was employed to further examine the mechanisms underlying cell death induced by combination therapy. As shown in Figure 4G,H, the Len@CMP and Len/FePt@CMP groups showed evidently downregulated expression of GPX4 and xCT protein (key markers of ferroptosis), due to the reaction of Len. Len/FePt@CMP NPs synergistically weaken antioxidant effects by regulating the production and consumption of GSH and GSSG within the cell. Accordingly, intracellular GSH and GSSG levels were quantitatively assessed using a Glutathione Detection Kit, revealing a substantial reduction in GSH content within the Len@CMP and Len/FePt@CMP groups, a substantial reduction in GSSG content within Len/FePt@CMP groups, as shown in Figure S5 (Supporting Information). Furthermore, the observed inhibition of FGFR4 protein by Len aligns with existing research, substantiating the assertion that it mediates its effects through the downregulation of the xCT‐GSH‐GPX4 pathway by inhibiting FGFR4. Besides, the expression of various apoptotic markers was examined via WB. The results illustrated that the treatment with FePt@CMP, Len@CMP and Len/FePt@CMP NPs upregulates the expression of pro‐apoptotic recombinant BCL‐2 associated X (BAX) and cleaved capspase‐3 proteins, while downregulating the anti‐apoptosis Bcl‐2 protein, which was ascribed to the pro‐apoptotic activity of FePt NPs and Len, providing additional therapeutic benefit (Figure 4I,J). Notably, Len/FePt@CMP NPs displayed the most pronounced pro‐apoptotic activity, highlighting its potent therapeutic efficacy.

### 2.5 Therapeutic Efficacy of Len/FePt@CMP NPs in HCC Mice

Encouraged by the promising therapeutic performance against HCCLM3 cells in vitro, the efficacy of Len/FePt@CMP NPs for the treatment of tumors in vivo was also evaluated. Initial studies were carried out in the subcutaneous HCC models, with the treatment protocol detailed in Figure S6A (Supporting Information). The tumor‐bearing mice were randomly divided into four groups, with each group receiving intravenous injections of PBS, FePt@CMP, Len@CMP, and Len/FePt@CMP NPs every three days for treatment, respectively. As depicted in the photographs of tumor‐bearing mice taken during the treatment (Figure S6B,C, Supporting Information), the mice in the Len/FePt@CMP group exhibited notably decreased tumor volumes, in contrast to the Len@CMP group with a slower tumor growth rate, while the FePt@CMP and PBS groups displayed rapid tumor growth. Quantitative tumor size assessments in the Len/FePt@CMP group indicated a consistent volumetric reduction over the treatment period, whereas tumors in the remaining groups displayed progressive growth, with the Len@CMP group exhibiting a moderated increase (Figure S6D, Supporting Information). Consequently, tumor weights in the Len/FePt@CMP group were significantly lower than those in the PBS, FePt@CMP, and Len@CMP groups (Figure S6E, Supporting Information). Furthermore, the survival rate of the mice in the Len/FePt@CMP group was higher than those in the other three groups (Figure S6F, Supporting Information). However, there were no significant differences observed in body weight of mice among the groups (Figure S6G, Supporting Information).

**Figure 5:**
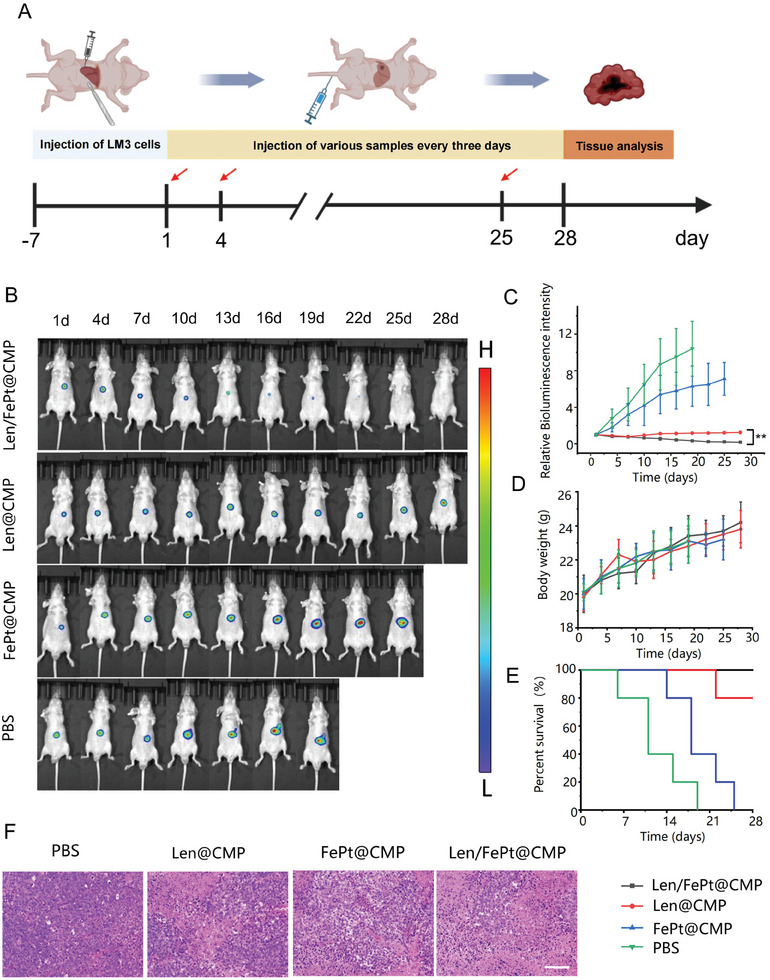
In vivo therapeutic efficacy of Len/FePt@CMP NPs in HCC tumor. A) Illustration of the treatment procedure. B) Bioluminescence images of tumor bearing mice under surveillance in each treatment group (*n* = 5). C) Quantification of tumor bioluminescence intensity. D) The body weight changes of mice in each treatment group (^**^ *p*‐value < 0.01). E) Survival in each group during treatment period. F) H&E staining of tumor tissue (scale bar: 200 µm).

Xenograft orthotopic HCC models employing HCCLM3‐luc cells (luciferase‐tagged HCCLM3 cells) were developed, offering a tumor growth milieu akin to clinical settings and rendering the tumor behavior more reflective of human conditions. The experimental design and treatment regimen for this model were aligned with those of the subcutaneous HCC studies (**Figure** **5**A). Tumor progression was monitored via bioluminescence intensity using the IVIS Imaging Spectrum System. Figure 5B,C indicated a progressive decline in bioluminescence within the Len/FePt@CMP group, in contrast to the escalation observed in the FePt@CMP and PBS groups, and stability within the Len@CMP group. Interestingly, it can be observed from the figure that tumors in the PBS group exhibited signs of metastasis in the terminal stage. Moreover, the body weight did not change significantly among the groups in the entire duration of the treatment (Figure 5D) and mice in the Len/FePt@CMP group had the longest survival period (Figure 5E). Tissues obtained from the tumors were subjected to hematoxylin and eosin (H&E) staining to elucidate alterations in the internal structures of tumors subjected to different treatment regimens, in which the Len/FePt@CMP group demonstrated the largest dead cell population (Figure 5F). These results further elucidated the extraordinary anti‐tumor effectiveness of Len/FePt@CMP NPs in vivo.

### 2.6 Validation of Enhanced Apoptosis and Ferroptosis in HCC Tumors

**Figure 6:**
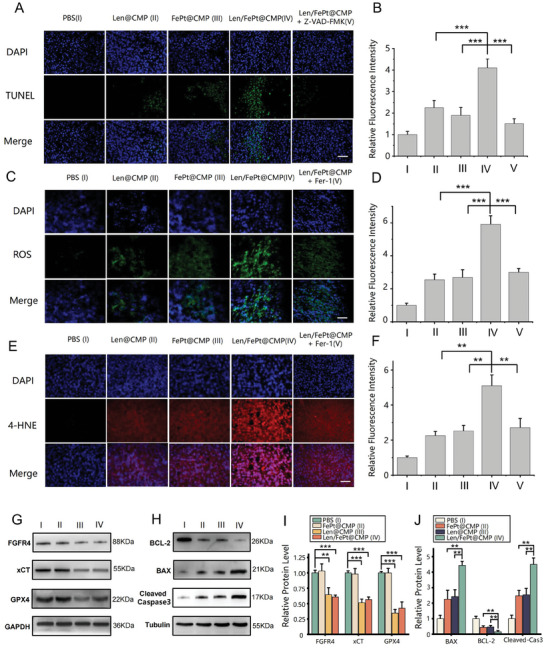
Enhanced ferroptosis and apoptosis effect in vivo. A) TUNEL staining of tumor tissue (scale bar: 50 µm). B) Quantification of fluorescence intensity analysis in A (^***^ *p*‐value < 0.001). C) ROS staining of tumor tissue (scale bar: 50 µm). D) Quantification of fluorescence intensity analysis in C (^***^ *p*‐value < 0.001). E) 4‐HNE staining of tumor tissue (scale bar: 50 µm). F) Quantification of fluorescence intensity analysis in E (^**^ *p*‐value < 0.01). G) WB analysis on the expression of key ferroptosis makers in tumor tissue with various treatments. H) WB analysis on the expression of key apoptosis makers in tumor tissue with various treatments. I) Quantitative measurement of ferroptosis‐associated proteins in G (^**^ *p*‐value < 0.01 and ^***^ *p*‐value < 0.001). J) Quantitative measurement of apoptosis‐associated proteins in H (^**^ *p*‐value < 0.01).

To further elucidate the enhanced apoptosis/ferroptosis phenomena and mechanisms in vivo, we employed the immunofluorescence technique to visualize tumor sections extracted from treated animals. As depicted in **Figure** **6**A,B, TUNEL staining of the Len/FePt@CMP group exhibited the most intense fluorescence, indicating the highest level of apoptosis, consistent with the H&E staining results. Additionally, the Len/FePt@CMP group demonstrated higher ROS levels compared to other groups, as depicted in Figure 6C,D. Notably, tumors treated with Len/FePt@CMP NPs exhibited the highest levels of 4‐hydroxynonenal (4‐HNE), a crucial class of reactive lipid species, suggesting that lipid peroxidation contributes to tumor cell death (Figure 6E,F). The differences of intratumoral ROS and 4‐HNE both uncovered a greater degree of oxidative stress in the Len/FePt@CMP group. Furthermore, the pathological alterations observed in the Len/FePt@CMP group were accompanied by reduced GPX4 activity (Figure S7, Supporting Information), establishing a link between ferroptosis and the functionality of crucial tumor suppressor pathways. To validate the involvement of tumor suppressor pathways, WB analysis of tumor tissues was conducted to examine protein expression changes following various treatments. The results revealed significant inhibition of FGFR4, xCT, and GPX4 protein expression in the tumor tissues of the Len@CMP and Len/FePt@CMP groups (Figure 6G,I), consistent with the inhibitory effect of Len and aligned with the in vitro findings. Furthermore, immunohistochemical staining of tumor tissue sections corroborated these results, further elucidating the mechanism through which Len/FePt@CMP NPs, in conjunction with Len@CMP NPs, induces ferroptosis by inhibiting the FGFR4‐xCT‐GSH‐GPX4 signaling pathway (Figure S8, Supporting Information). Additionally, WB analysis of apoptosis markers revealed the most increased expression of pro‐apoptotic BAX and cleaved caspase‐3 protein and decreased expression of anti‐apoptotic Bcl‐2 protein in the Len/FePt@CMP group, consistent with the outcomes of in vitro research (Figure 6H,J). These comprehensive experimental results support the hypothesis that Len/FePt@CMP NPs effectively inhibits tumor growth in vivo due to the enhanced the dual effect of ferroptosis and apoptosis.

### 2.7 Tumor mRNA Analysis of Enhanced Apoptosis and Ferroptosis Effect

**Figure 7:**
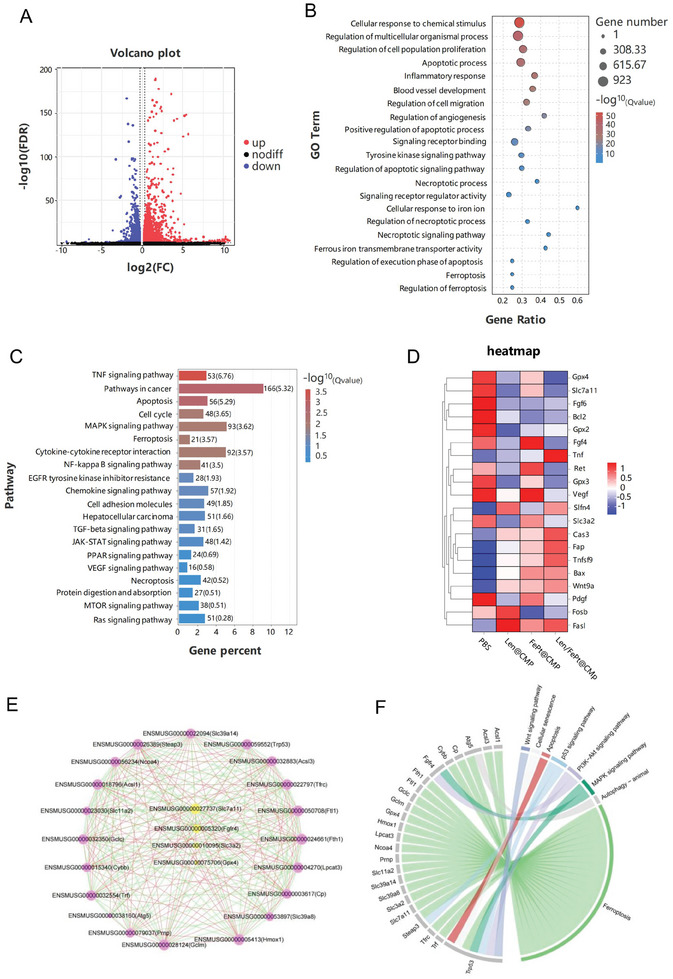
mRNA sequence analysis of tumor tissue. A) Volcano plot of gene expression of upregulated and downregulated genes between PBS and Len/FePt@CMP groups (log2FC ≥ 1.5, FDR ≤ 0.5). B) GO enrichment analysis of DEGs in Len/FePt@CMP group. C) KEGG pathways enrichment analysis of DEGs in Len/FePt@CMP group. D) A heatmap analysis of apoptosis/ferroptosis‐related DEGs among different treatment groups. E) PPI network analysis of specific gene interaction pathways derived from representative DEGs. F) The Circos plot visualization of significant associations between representative DEGs and KEGG pathways.

To demonstrate pathways and mechanisms of Len/FePt@CMP NPs action in vivo, the RNA sequencing analysis of HCC tumor with different treatments was executed. The distribution of differentially expressed genes (DEGs) was depicted in Figure S9 (Supporting Information), with the Len/FePt@CMP group exhibiting the most significant variation in the number of DEGs. In comparison to the PBS group, 2 053 DEGs were upregulated while 1 865 DEGs were downregulated in the Len/FePt@CMP group (**Figure** **7**A). Gene ontology (GO) enrichment analysis revealed that the expression of DEGs in response to Len/FePt@CMP NPs treatment was associated with apoptotic processes, positive regulation of apoptotic processes, regulation of apoptotic signaling pathways, cellular response to iron ions, regulation of the execution phase of apoptosis, regulation of necroptotic processes, and regulation of ferroptosis, underscoring the pivotal roles of ferroptosis and apoptosis in the therapeutic process (Figure 7B). Furthermore, Kyoto Encyclopedia of Genes and Genomes (KEGG) pathway enrichment analysis unveiled the correlated pathways of DEGs regulated by Len/FePt@CMP NPs treatment, including the tumor necrosis factor (TNF) signaling pathway, apoptosis, mitogen‐activated protein kinases (MAPK) signaling pathway, ferroptosis, NF‐kappa B signaling pathway, and others (Figure 7C). Additionally, a correlation heatmap was generated to illustrate the distinct expression patterns of 20 representative DEGs among the various treatment groups (Figure 7D), in which, the red indicates upregulation and blue indicates downregulation. The expression patterns of genes such as BAX, Bcl‐2, cleaved caspase‐3, FGFR4, xCT, and GPX4, were consistent with the protein expression results obtained from WB analysis. To delineate the interconnections among these representative genes, a protein‐protein interaction (PPI) network was constructed to illustrate the relationships encoded by these genes. The PPI results indicated that FGFR4, GPX4, and xCT, associated with the ferroptosis pathway, are critical genes in the treatment of HCC (Figure 7E). Then, we presented a Circos plot to visualize the relationships between representative DEGs and pathways. It revealed that the largest number of representative DEGs is enriched in the ferroptosis pathway (Figure 7F), providing further evidence for Len triggering ferroptosis through the FGFR4‐xCT‐GSH‐GPX4 signaling pathway. It was reported that activating mutations of phosphatidylinositol 3‐kinase (PI3K) conferred ferroptosis resistance in cancer cells and that inhibition of the PI3K‐AKT‐mechanistic target of rapamycin signaling axis sensitized cancer cells to ferroptosis induction.^[^ ^27^ ^]^ Some reports have shown that activated FGFR4 directly phosphorylated FGFR substrate 2, which led to the activation of PI3K‐AKT signaling.^[^ ^28^ ^]^ It is possible that the inhibition of FGFR4 by Len suppressed the activation of PI3K‐AKT signaling, thereby inducing ferroptosis. In conclusion, a considerable number of DEGs were identified following Len/FePt@CMP NPs treatment, which induced transcriptional dysregulation and affecting oncogenic signaling in HCC, particularly in apoptosis and ferroptosis.

### 2.8 Biosafety Evaluation of Len/FePt@CMP NPs

A series of biotoxicity experiements were employed to assess the biosafety of Len/FePt@CMP NPs. Hemolysis rates were measured following the incubation of blood samples with different concentrations of Len/FePt@CMP NPs. The results demonstrated no significant evidence of hemolysis even at a high concentration of 300 µg mL^−1^, indicating the safety of intravenous injection of Len/FePt@CMP NPs (Figure S10A, Supporting Information). Then, we employed the CCK‐8 assay to evaluate the cytotoxicity of Len/FePt@CMP NPs on normal liver cells (LO2) in vitro. The results showed that cell viability remained above 80% under high concentration (Figure S10B, Supporting Information). This outcome can be attributed to the weaker uptake of cancer cell membrane coated NPs by normal liver cells. Moreover, the lower concentration of hydrogen peroxide in normal liver cells is insufficient to trigger an effective Fenton reaction. Further assessment of in vivo toxicity was conducted through blood biochemistry parameters and histological analyses of major organs after various treatments. Pathological sections of major organs revealed no noticeable signs of inflammation or tissue damage (Figure S10C, Supporting Information). Additionally, all blood biochemistry parameters remained within the normal range in treated groups compared to the PBS group, with no obvious differences observed (Figure S10D–I, Supporting Information). These findings underscore the excellent biological safety and biocompatibility of Len/FePt@CMP NPs, which extend its potential for clinical translation.

## 3 Conclusion

In summary, our study introduces a novel intracellular remodeling strategy for co‐delivery of FePt NPs and Len using HCC cell membrane‐coated PLGA NPs, which effectively initiates targeted and enhanced dual ferroptosis/apoptosis actions for efficient tumor inhibition. We have validated that Len inhibits xCT and inactivates GPX4 by suppressing FGFR4, resulting in accumulation of LPO and ROS. This mechanism of Len‐induced ferroptosis has not been sufficiently studied in previous research, providing an opportunity to improve the efficacy of Len. Both in vitro and in vivo evidence collectively demonstrate that the ferroptosis sensitizing ability of Len, in cooperation with enriched Fe^2+^ released by FePt NPs, elicits substantial ferroptotic, and apoptotic damage to HCC tumor. This enhanced ferroptosis and apoptosis treatment efficacy, triggered by Len/FePt@CMP NPs, is superior to that of any single therapy. Furthermore, the biomimetic modification of NPs with HCC cell membrane achieves accurate homologous targeting and immune evasion for treating HCC, while ensuring excellent biosafety. Additionally, we noninvasively monitored the accumulation and metabolism of Len/FePt@CMP NPs at the tumor site using PAI due to the remarkable contrast‐enhancing capacities of FePt NPs. This approach can guide the timing of drug therapy and holds potential for future applications in early tumor diagnosis and drug efficacy monitoring. In conclusion, developing new mechanisms and therapeutic strategies based on clinical needs is the inexorable trend in current research. Our mentality of designing has potential in offering novel pathways and prospects for treating HCC in clinical setting.

## 4 Experimental Section

### Synthesis of FePt NPs

FePt NPs were synthesized using a thermo‐reduction procedure. In a three‐necked flask, 290 mg of platinum acetylacetone was combined with 353 mg of iron acetylacetone, 774 mg of 1,2‐hexadecandiol, 4 mL of oleic acid, 4 mL of oleylamine, and 4 mL of dioctyl ether. The mixture was then heated to 100^ °^C at a heating rate 15^ °^C min^−1^ under the protection of a gentle N_2_ flow. After 20 min, the reaction system was further heated to 240^ °^C at a rate of 15^ °^C min^−1^ and maintained for 1 h before being cooled to room temperature. The resultant product was washed three times with chloroform and ethanol before being stored in chloroform. After freeze‐drying, FePt NPs were stored in N_2_ atmosphere for further use.

### Preparation of Drug‐Loaded PLGA NPs

To prepare the dual‐loaded PLGA NPs, 10 mg of Len, 10 mg of FePt, and 300 mg of PLGA (50 ∶ 50) were dissolved in 10 mL of dichloromethane under sonication to prepare the oil phase. The oil phase was then mixed with 1 mL of double‐distilled water (ddH_2_O) as the water phase and subjected to ultrasonic wave treatment (Kunshan KQ250, China) for 10 min. Subsequently, the mixed emulsion was rapidly added to 100 mL of 2% polyvinyl alcohol (PVA) solution under high‐speed magnetic stirring to form microspheres. Finally, the microspheres were magnetically stirred at 800 rpm for 8 h at room temperature to completely evaporate the dichloromethane solvent. The resultant sample (Len/FePt@PLGA NPs) was frozen for 12 h after being washed three times with ddH_2_O and then vacuum‐dried (Christ Freeze dryer, Germany) for subsequent use. Cy5/Len/FePt@PLGA, Len@PLGA and FePt@PLGA NPs were prepared using the same method.

### Cell Membrane Extraction

To isolate membranes, HCCLM3 cells were cultured in T75 flasks until they reached full confluency. The cells were detached using 2 mm EDTA, followed by three washes in PBS through centrifugation at 800 g for 5 min. Subsequently, the cells were suspended in a hypotonic lysis buffer comprising 20 mm Tris‐HCl, 10 mm KCl, 2 mm MgCl_2_, and one EDTA‐free mini protease inhibitor tablet per 10 mL of solution. Disruption was achieved using a Dounce homogenizer with a tightly fitting pestle, performing 20 passes. The entire solution was centrifuged at 3 200 g for 5 min, saving the supernatants. The pellet was then resuspended in the hypotonic lysis buffer, subjected to another 20 passes, and spun down again. The supernatants from the repeated passes were pooled and centrifuged at 20 000 g for 30 min, followed by discarding the pellet. The remaining supernatant underwent another centrifugation at 80 000 g for 1.5 h using an ultra‐speed centrifuge (LE‐80K, Beckman Coulter, USA). The pellet, containing purified cancer cell membranes, was subsequently washed once with 10 mm Tris‐HCl and 1 mm EDTA before being collected as the final purified membrane pellet.

### Preparation of Len/FePt@CMP NPs

For the encapsulation of cancer cell membranes onto Len/FePt@PLGA NPs, a solution consisting of 1 mL PBS with 50 µg Len/FePt@PLGA NPs was combined with the previously prepared cancer cell membranes (mass of membrane‐to‐core ratio greater than 20∶1). The mixture underwent extrusion 11 times through 200nm polycarbonate porous membrane on a mini extruder, followed by centrifugation at 1000 g to eliminate surplus cancer cell membranes. Subsequently, the resulting nanoparticles, coated with cancer cell membranes (Len/FePt@CMP NPs), were incubated in PBS overnight at 4^ °^C for subsequent applications. Cy5/Len/FePt@CMP, Len@CMP and FePt@CMP NPs were synthesized using the same method.

### Characterization of Len/FePt@CMP NPs

The morphologies FePt NPs and Len/FePt@CMP NPs were investigated via transmission electron microscopy (TEM, JEM‐F200, Japan). The hydrodynamic diameters and zeta potential of NPs were measured using Zetasizer Nano (ZS) (Malvern Instruments, UK). Len in Len/FePt@CMP NPs was quantified using HPLC (Shimadzu, Japan). In addition, the stability of Len/FePt@CMP NPs was assessed by measuring size changes following incubation in 1 × PBS or PBS containing 10% FBS at 0, 2, 4, 8, 12, 24, 36, and 48h. The nanoparticles were destroyed and Len was dissolved by methanol. Analytes were run on a reversed‐phase C18 column (Ultimate Plus‐C18 4.6×150 mm 5µm) followed by specific measurements at 220 nm. The encapsulation efficiency (EE%) and drug loading (DL%) were calculated based on the HPLC results and the formulas were as follows:

(1) $$\mathrm{EE}\%\;=\left[\mathrm{weight}\;\mathrm{of}\;\mathrm{Len}\;\mathrm{in}\;\mathrm{NPs}\;/\;\mathrm{weight}\;\mathrm{of}\;\mathrm{Len}\;\mathrm{in}\;\mathrm{feed}\right]\times 100\%$$

(2) $$\mathrm{DL}\%\;=\left[\mathrm{weight}\;\mathrm{of}\;\mathrm{Len}\;\mathrm{in}\;\mathrm{NPs}\;/\;\mathrm{weight}\;\mathrm{of}\;\mathrm{NPs}\right]\times 100\%$$

FePt NPs in Len/FePt@CMP NPs were quantified by a SPECTROBLUE FMX36 inductively coupled plasma optical emission spectrometer (ICP‐OES, SPECTRO, Germany). SDS‐PAGE analysis was performed to analyze protein profiles of HCCLM3 cell membrane, Len/FePt@CMP NPs, and Len/FePt@PLGA NPs. The proteins were stained with Coomassieblue (Invitrogen) and imaged after destaining in water overnight. The optical absorbance spectra of Len/FePt@CMP NPs was measured by microplate reader (Biotek, USA). Gradient concentrations of Len/FePt@CMP NPs dispersions (12.5, 25, 50, 100, and 200 µg mL^−1^) were assessed for in vitro PAI performance using a Vevo 3100 animal PAI system (FUJIFILM VisualSonics, Japan).

### In Vitro Drug Release Study

Len/FePt@CMP and Len/FePt@PLGA NPs (1 mL, 1 mg mL^−1^) loaded in dialysis bags (MW∶3500 Da) were immersed in 20 mL of PBS and incubated at 37^ °^C and pH 6.5. At predetermined time points (0.5, 1, 2, 4, 8, 12, and 24 h), 0.2 mL of the medium was drawn out and added with an equal volume of fresh medium. Then, the Len concentration was determined using HPLC analysis and the FePt concentration was detected using ICP‐OES. Repeat the above experiment under pH 7.4 conditions, maintaining the same steps.

### OH Generation of Len/FePt@CMP In Vitro

PBS (pH = 5) solutions of MB (10 µg mL^−1^) containing Len/FePt@CMP NPs (20 µg mL^−1^) and different concentrations of H_2_O_2_ (0, 10, 20, and 30 mm) were incubated in a 37^ °^C aqueous bath for 30 min. Subsequently, the absorbance changes of MB were measured by microplate reader. To investigate the influence of Len/FePt@CMP NPs concentration on the production of ·OH, the absorbance of MB in PBS (pH 5) treated with gradient Len/FePt@CMP NPs concentrations (0, 10, 20, and 40 µg mL^−1^) and H_2_O_2_ (30 mm) was measured. To investigate the influence of pH values on the formation of ·OH, MB was incubated with Len/FePt@CMP NPs (20 µg mL^−1^) and H_2_O_2_ (30 mm) in different pH values (7, 6, 5, and 4).

### Cell Culture Experiment

Hepatocellular carcinoma cells (HCCLM3 and HepG2), hepatic cells (LO2), lung cancer cells (A549), Breast cancer cells (4T1), and macrophage RAW264.7 cells were cultured in high‐glucose Dulbecco's Modified Eagle Medium (DMEM, Gibco), supplemented with 10% fetal bovine serum (FBS, Gibco) and 1% penicillin/streptomycin (pen/strep, Gibco) in a 37^ °^C humidified incubator (Thermo scientific, USA) with 5% CO2. All cell lines were purchased from Procell (China).

### In Vitro Generation of 3D Tumor Spheroids

HCCLM3 cells were seeded at a density of 6 000 cells per well in 96‐well Corning Costar ultra‐low attachment round‐bottom plates (Corning Incorporated, USA) and cultured for five days to allow for spheroid formation. Spheroids with a diameter of ≈400 µm were considered suitable for experiment use.

### In Vitro Specificity Evaluation Study

The Len/FePt@CMP NPs were labeled with Cy5 and loaded into the hydrophobic inner core of the particles, following the same method used for preparation of Drug‐Loaded PLGA NPs. PBS, Cy5/Len/FePt@CMP NPs (0.1 mg mL^−1^), and Cy5/Len/FePt@PLGA NPs (0.1 mg mL^−1^) were co‐cultured with RAW264.7 cells for 4 h to assess macrophage phagocytosis resistance and then washed with PBS three times, fixed with 4% paraformaldehyde for 30 min, and subjected to nuclear staining with DAPI for 10 min. Finally, the cells were washed with PBS three times again and then examined using a fluorescence microscope (Nikon, Japan).

Cy5/Len/FePt@CMP NPs (0.1 mg mL^−1^) was co‐cultured with various cell lines for 4 h, including RAW264.7 cells to assess macrophage phagocytosis resistance, and HCCLM3, HepG2 liver cancer cells, A549 lung cancer cells, and 4T1 breast cancer cells to evaluate its specific targeting ability. After the incubation period, cells were washed with PBS three times, fixed with 4% paraformaldehyde for 30 min, and subjected to nuclear staining with DAPI for 10 min. Finally, the cells were washed with PBS three times again and then examined using a fluorescence microscope.

To verify the endocellular Fe^2+^, various cells (HCCLM3, HepG2, A549, and 4T1) were subjected to co‐incubation with Len/FePt@CMP NPs (0.1 mg mL^−1^) for 4 h. Later, the cells were stained with FerroOrange (Dojindo, Japan) for 30 min and observed under a fluorescence microscope.

### In Vitro Cytotoxicity

HCCLM3 and LO2 cells were seeded in 96‐well plates (5000 cells in 100 µL medium per well) and incubated for 24 h to allow their adherence. The cells were then incubated with Len/FePt@CMP, Len@CMP, and FePt@CMP NPs at gradient concentrations for another 24 h. The cell viability was measured with CCK‐8 assay. The measurements were based on absorbance at 450 nm using a microplate reader.

HCCLM3 cells were seeded in 96‐well plates (5000 cells in 100 µL medium per well) and incubated for 24 h to allow their adherence. The cells were divided into five groups and incubated with PBS, Len/FePt@CMP (15 µg mL^−1^), Len/FePt@CMP (15 µg mL^−1^)+Fer‐1 (5 µm), Len/FePt@CMP (15 µg mL^−1^)+Z‐VAD‐FMK (50 µm), and Len/FePt@CMP (15 µg mL^−1^)+Fer‐1 (5 µm)+Z‐VAD‐FMK (50 µm) for another 24 h. The cell viability was measured with CCK‐8 assay. The measurements were based on absorbance at 450 nm using a microplate reader.

The tumor spheroids were divided into four groups and treated with PBS, Len/FePt@CMP (0.1 mg mL^−1^), Len@CMP (0.1 mg mL^−1^), and FePt@CMP NPs (0.1 mg mL^−1^), respectively. The culture medium containing PBS, Len/FePt@CMP, Len@CMP, and FePt@CMP NPs was replaced every three days, and changes in size and morphology were monitored under a microscope.

### Live/Dead Cell Staining Assays

HCCLM3 cells were seeded at a density of 6000 cells per well in 96‐well Corning Costar ultra‐low attachment round‐bottom plates and then allowed to grow until the spheroid diameter reached approximately 600 µm. The medium was then replaced with fresh medium containing PBS, Len/FePt@CMP (0.1 mg mL^−1^), Len@CMP (0.1 mg mL^−1^), and FePt@CMP NPs (0.1 mg mL^−1^) and the cells were subsequently incubated for an additional 24 h. Next, the cells were co‐stained with Calcein‐AM and propidium iodide (PI) for 1 h, washed twice with PBS, and visualized under a fluorescence microscope.

### Apoptosis Assay by Flow Cytometry

HCCLM3 cells were cultured in a 12‐well plate (10 000 cells in 1 mL medium per well) for 24 h. The medium was then replaced with fresh medium containing PBS, Len/FePt@CMP (0.1 mg mL^−1^), Len@CMP (0.1 mg mL^−1^), FePt@CMP NPs (0.1 mg mL^−1^), and FePt@CMP NPs (0.1 mg mL^−1^)+Z‐VAD‐FMK (50 µm) and the cells were subsequently incubated for an additional 6 h. The cells were collected and washed with PBS three times then resuspended in 100 µL 1× annexin binding buffer. The harvested cells were counterstained with 100 µg mL^−1^ propidium iodide (PI) and FITC labeled annexin V for 15 min, and the stained cells were subjected to flow cytometry analysis (Beckman Coulter, USA).

### Intracellular ROS Induction

HCCLM3 cells were cultured in a 12‐well plate (5000 cells in 1 mL medium per well) for 24 h. The medium was then replaced with fresh medium containing PBS, Len/FePt@CMP (0.1 mg mL^−1^), Len@CMP (0.1 mg mL^−1^), FePt@CMP NPs (0.1 mg mL^−1^), and FePt@CMP NPs (0.1 mg mL^−1^)+Fer‐1 (5 µm) and the cells were subsequently incubated for an additional 4 h. After incubating, the cells were washed with PBS three times and stained with DCFH‐DA (Solarbio, China) for 30 min. The stained cells were then collected, washed three times with PBS, and subjected to flow cytometry analysis.

### Lipid Peroxides Fluorescence Staining

HCCLM3 cells were cultured in a 12‐well plate (5000 cells in 1 mL medium per well) for 24 h. The medium was then replaced with fresh medium containing PBS, Len/FePt@CMP (0.1 mg mL^−1^), Len@CMP (0.1 mg mL^−1^), FePt@CMP NPs (0.1 mg mL^−1^), and FePt@CMP NPs (0.1 mg mL^−1^)+Fer‐1 (5 µm) and the cells were subsequently incubated for an additional 6 h. The cells were stained with BODIPY581/591‐C11 (Invitrogen, USA) for 30 min, washed twice with PBS, and observed under a fluorescence microscope.

### Monitoring the Changes in Mitochondrial Membrane Potential

HCCLM3 cells were cultured in a 12‐well plate (5000 cells in 1 mL medium per well) for 24 h. The medium was then replaced with fresh medium containing PBS, Len/FePt@CMP (0.1 mg mL^−1^), Len@CMP (0.1 mg mL^−1^), FePt@CMP NPs (0.1 mg mL^−1^) and FePt@CMP NPs (0.1 mg mL^−1^)+Fer‐1 (5 µm) and the cells were subsequently incubated for an additional 8 h. The cells were stained with JC‐1 dye following the procedures provided in the user manual and observed under a fluorescence microscope.

### Observation of Subcellular Changes using TEM

HCCLM3 cells were cultured in a 6‐well plate (20 000 cells in 2 mL medium per well) for 24 h. Then the medium was replaced with fresh medium containing PBS, Len/FePt@CMP (0.1 mg mL^−1^), Len@CMP (0.1 mg mL^−1^), FePt@CMP NPs (0.1 mg mL^−1^), and FePt@CMP NPs (0.1 mg mL^−1^)+Fer‐1 (5 µm) and the cells were subsequently incubated for an additional 24 h. Cells were collected using a cell scraper, then centrifuged at 2500 g for 3 min to form cell pellets. Finally, cell pellets were fixed with 2.5% glutaraldehyde solution and fix at 4^ °^C for 12 h. The fixed cell pellets were sliced and observed under the TEM.

### Western Blot Analysis

HCCLM3 cells were cultured in a 6‐well plate (20 000 cells in 2 mL medium per well) for 24 h. Subsequently, the medium was replaced with fresh medium containing PBS, Len/FePt@CMP (0.1 mg mL^−1^), Len@CMP (0.1 mg mL^−1^), and FePt@CMP NPs (0.1 mg mL^−1^) and the cells were subsequently incubated for an additional 24 h. The protein was extracted using 10‐fold RIPA buffer (Solarbio, China), lysed on ice bath for 30 min, and measured by BCA protein assay kit (Beyotime, China). Then protein samples were diluted with a running buffer to 2.5 mg mL^−1^ and heated for 10 min in boiling water. Subsequently, proteins in 10 µL samples were separated by Bis‐Tri's gel. For WB, the gel was blotted onto a PVDF membrane by wet transfer. Then the membrane was washed with 0.25% Tween20 Tris‐buffered saline (TBST) and blocked with 5% milk for 2 h. The membrane was cut according to the marker and overnight incubated at 4^ °^C with primary antibodies (Abcam, USA) including FGFR4, xCT, GPX4, GAPDH, Cleaved Caspase3, BAX, BCL‐2, and Tubulin. Subsequently, the membrane was washed with TBST three times and incubated with second antibodies. After incubating with second antibodies at room temperature for 1 h and washed with TBST three times, the membrane was stained with ECL chemiluminescence reagents and photographed for analysis (Bio‐Rad, USA). Western blot assays for tumor tissues were performed the same as cells.

### Detection of Intracellular GSH and GSSG Level

HCCLM3 cells were cultured in a 6‐well plate (20 000 cells in 2 mL medium per well) for 24 h. Then the cells were incubated with Len/FePt@CMP, Len@CMP, and FePt@CMP NPs at gradient concentrations for another 24 h. Following the incubation period, cells were washed three times with PBS buffer and then collected by centrifugation. Subsequently, protein lysis buffer was added to the cell pellet in an amount equal to three times its volume. Next, the samples were subjected to two cycles of rapid freeze‐thaw and centrifuged at 10 000 g at 4^ °^C for 10 min and the supernatant was collected. A portion of the prepared samples was taken and diluted GSH scavenging auxiliary solution was added at a ratio of 20 µL per 100 µL of sample. The mixture was vortexed immediately to mix well. Then, GSH scavenging working solution was added at a ratio of 4 µL per 100 µL of sample. The mixture was vortexed immediately to mix well and allowed to react at 25^ °^C for 60 min. Sequentially, standards and all test samples were added to a 96‐well plate, followed by the addition of 150 µL of total glutathione detection working solution per well. After incubation at room temperature for 5 min, 50 µL of prepared NADPH solution was added per well, ensuring thorough mixing, and the absorbance was measured at 405–414 nm using a microplate reader. Finally, the concentrations of GSH and GSSG were calculated based on the absorbance and the standard curve.

### Tumor Model Establishment

To establish the subcutaneous hepatocellular carcinoma model, ≈1 × 10^6^ HCCLM3 cells (100 µL PBS) were injected into the right hind limb dorsal region of BALB/c nude mice (5‐week‐old, male). The establishment of the other subcutaneous models with A549, HepG2, and 4T1 cells were performed same as this method.

To generate the orthotropic xenograft model, BALB/c nude mouse (5‐week‐old, male) were anesthetized using inhalation of isoflurane. The mouse was fixed in a supine position, and the layers of skin and peritoneum were cut at approximately 0.8 cm below the xiphoid line at the ventral midline, followed by implantation of cell suspensions containing 1 × 10^6^ HCCLM3‐luc cells and Matrigel at a 1∶1 (v/v) ratio into the left lobe of the liver. The peritoneum and skin were sutured using 6‐0 silk. To assess tumor growth, a small animal optical molecular imaging system (IVIS Imaging Spectrum System, PerkinElmer, USA) was used after 7 days. The bioluminescent signal of orthotropic xenograft liver cancer was generated by the interaction of luciferase from HCCLM3 cells with D‐luciferin solution (15 mg mL^−1^), which was injected into the abdomen before imaging.

All mice were purchased from Zhuhai Baicutong Biotechnology Co., LTD (China) and raised in standard laboratory conditions of Zhujiang hospital with a 12 ∶ 12‐h light/dark cycle and provided free access to food and water.

### In Vivo Fluorescence and PA Imaging

To verify the homologous targeting ability of Len/FePt@CMP NPs in vivo, HCCLM3, A549, HepG2, and 4T1 subcutaneous tumor bearing mice (*n* = 3) were intravenously injected with Cy5/Len/FePt@CMP NPs (0.8 mg mL^−1^, 200 µL). Fluorescence images were acquired at pre‐determined time points (before, 2, 6, 12, and 24 h) using an in vivo IVIS Imaging Spectrum System.

To investigate the aggregation of NPs within the tumor, HCCLM3 subcutaneous tumor bearing mice were randomly divided into three groups (*n* = 3) and intravenously injected with PBS (200 µL), Len/FePt@PLGA (0.8 mg mL^−1^ based on the weight of FePt NPs concentration, 200 µL), and Len/FePt@CMP (0.8 mg mL^−1^ based on the weight of FePt NPs concentration, 200 µL). PA images of the tumor cross‐sections were acquired at different time points (before, 2, 6, 12, and 24 h) using a Vevo 3100 animal PAI system.

### Blood Circulation Time Analysis

The blood samples of mice in different groups were collected from tail vein at 0.5, 1, 2, 6, 12, and 24 h after the injection of the nanoprobes. The blood was thoroughly mixed with the anticoagulant and methanol, the supernatant was obtained after centrifugal force at 3000 rpm for 10 min. Analytes were run on a reversed‐phase C18 column (Ultimate Plus‐C18 4.6 ×150 mm 5 µm) followed by specific measurements at 220 nm to detect the Len concentration in blood.

### Len/FePt@CMP NPs Distribution Analysis

The organ and tumor samples of mice were extracted from different groups after PA imaging. After degradation by aqua regia and filtration through 0.22 µm polyether sulfone membranes, the FePt NPs concentration of the samples were quantified by ICP‐OES.

### In Vivo Therapy of Len/FePt@CMP NPs

HCCLM3 subcutaneous tumor bearing mice were randomly divided into four groups (*n* = 5) and intravenously injected with PBS, FePt@CMP (6.5 mg kg^−1^ based on the weight of FePt NPs concentration), Len@CMP (10 mg kg^−1^ based on the weight of Len concentration), and Len/FePt@CMP NPs (10 mg kg^−1^ based on the weight of Len concentration) every three days for treatment, respectively. Tumor size and weight were monitored every three days after treatment in each group. Tumor volume (V) was determined using the formula: V  =  length  ×  width^2^/2 (mm^3^). Mice with tumors larger than ≈1000 mm^3^ were euthanized according to the standard animal protocol.

HCCLM3 orthotropic tumor bearing mice were randomly divided into four groups (*n* = 5) and intravenously injected with PBS, FePt@CMP (6.5 mg kg^−1^ based on the weight of FePt NPs concentration), Len@CMP (10 mg kg^−1^ based on the weight of Len concentration), and Len/FePt@CMP NPs (10 mg kg^−1^ based on the weight of Len concentration) every three days for treatment, respectively. The bioluminescent signal of orthotropic xenograft HCC was monitored using an IVIS Imaging Spectrum System. The treatment effect on each group was determined by measuring the body weight, survival, and tumor signal intensity. After completing the treatment period, all mice were humanely euthanized and their tumors were harvested. The tumors were rinsed with PBS, fixed in 4% paraformaldehyde and subsequently embedded in paraffin. Paraffin‐embedded tumor sections were prepared using a microtome (Leica RM2235, Germany) and affixed to slides. Standard histological procedures, including H&E staining, as well as immunohistochemical staining for FGFR4, GPX4, and xCT, were conducted on separate sections. The sections were then visualized using a virtual slide microscope (Olympus VS120, Japan).

HCCLM3 orthotropic tumor bearing mice were randomly divided into six groups (*n* = 3) and intravenously injected with PBS, FePt@CMP (6.5 mg kg^−1^ based on the weight of FePt NPs concentration), Len@CMP (10 mg kg^−1^ based on the weight of Len concentration), Len/FePt@CMP NPs (10 mg kg^−1^ based on the weight of Len concentration), Len/FePt@CMP NPs (10 mg kg^−1^ based on the weight of Len concentration)+Fer‐1 (2 mg kg^−1^), and Len/FePt@CMP NPs (10 mg kg^−1^ based on the weight of Len concentration)+Z‐VAD‐FMK (5 mg kg^−1^) once daily for three consecutive days, respectively. Then all mice were humanely euthanized and their tumors were harvested. The tumors were rinsed with PBS, fixed in 4% paraformaldehyde and subsequently embedded in paraffin. Paraffin‐embedded tumor sections were prepared using a microtome and affixed to slides. Standard histological procedures, including TUNEL, ROS, and 4‐HNE immunofluorescence staining, were conducted on separate sections. The sections were then visualized using a virtual slide microscope.

### Transcriptomic mRNA Sequencing Analysis

Total RNA was extracted using the Trizol reagent kit (Invitrogen, USA) following the manufacturer's instructions. The RNA quality was assessed on an Agilent 2100 Bioanalyzer (Agilent Technologies, USA) and verified using RNase‐free agarose gel electrophoresis. Following total RNA extraction, eukaryotic mRNA was enriched using Oligo(dT) beads (Epicentre, USA). The enriched mRNA was then fragmented into short fragments using fragmentation buffer and reverse transcribed into cDNA using the NEBNext Ultra RNA Library Prep Kit for Illumina (NEB #7530, USA). The purified double‐stranded cDNA fragments underwent end repair, addition of an A base, and ligation to Illumina sequencing adapters. The ligation reaction was purified with AMPure XP Beads (1.0X), followed by polymerase chain reaction (PCR) amplification. The resulting cDNA library was sequenced using Illumina Novaseq6000. RNAs differential expression analysis was performed by DESeq2 software among different treated groups. The genes/transcripts with the parameter of false discovery rate (FDR) below 0.05 and absolute fold change (log_2_FC) ≥ 2 was considered differentially expressed genes/transcripts. The gene ontology (GO) enrichment analysis, pathway enrichment analysis, protein‐protein interaction analysis and DEGs‐pathways interaction analysis were further performed and visualized by DESeq2 software.

### Biosafety Evaluation

Hemolysis analysis: Fresh blood (1.0 mL) from BALB/c nude mice were collected into an anticoagulation tube containing ethylenediaminetetraacetic acid (EDTA) with an addition of 2 mL PBS. After centrifugation (3 000 rpm, 10 min, 4^ °^C) and three washes with PBS, the obtained red blood cells (RBCs) were dispersed in 10 mL PBS. Subsequently, the RBC dispersions (0.2 mL) were gently mixed and incubated with 0.8 mL DI water (positive control), and Len/FePt@CMP NPs dispersions at different concentrations (0, 50, 100, 150, 200, 250, and 300 µg mL^−1^) at 37^ °^C for 3 h. The samples were then centrifuged (3000 rpm, 10 min) and the supernatants were collected for absorbance measurements at 577 nm. Finally, the hemolysis percentage of each sample was calculated.

After completing the treatment period, the blood samples and the main organs of various treated groups (PBS, Len@CMP NPs, FePt@CMP NPs, and Len/FePt@CMP NPs) were harvested for biosafety evaluation. The blood samples were centrifugated at 4^ °^C and 6 000 rpm for 10 min to obtain the serum. The blood biochemical parameters including alanine aminotransferase (ALT), aspartate aminotransferase (AST), alkaline phosphatase (ALP), albumin (ALB), lactate dehydrogenase (LDH), and uric acid (UA) were tested. These extracted organs were fixed in 4% paraformaldehyde and subjected to H&E staining for histological analysis.

### Statistical Analysis

SPSS Statistic 27 was used for the data analyses. All data were expressed as the mean ± standard error. A two‐tailed paired Student's *t*‐test was used to compare the differences. ^*^ *p*‐value < 0.05, ^**^ *p*‐value < 0.01, and ^***^ *p*‐value < 0.001 were considered statistically significant.

### Ethics Approval Statement

All animal procedures were approved by the Zhujiang Hospital Animal Welfare Ethics Review Committee and were conducted in accordance with national regulations and protocols (Ethics No. LAEC‐2022‐199).

## Conflict of Interest

The authors declare no conflict of interest.

## Author Contributions

F. X. and X. Z. contributed equally to this work. F. X. and X. Z. contributed to designing, performing, and analyzing experiments; writing the manuscript. Z. L. and X. Y. contributed to performing part of experiments and analyzing the experimental data. W. X. and X. Z. contributed to collecting and analyzing experimental data. L. N. contributed to providing experiment facilities. J. Y., S. L., and W. P.contributed to reviewing and polishing the manuscript. P. L. and C. F. contributed to supervising and coordinating the project; reviewing and revising the manuscript. All authors read and approved the final manuscript.

## Supporting information



Supporting Information

## Data Availability

Research data are not shared.
