# Supplementary material for: Biomimetic Co‐delivery of Lenvatinib and FePt Nanoparticles for Enhanced Ferroptosis/Apoptosis Treatment of Hepatocellular Carcinoma
Source: Adv Healthc Mater. 2025 Mar 21;14(11):2401747. doi: 10.1002/adhm.202401747 (PMC12023810; doi:10.1002/adhm.202401747)
Supplement: Supplementary file 1 — Supporting Information [file ADHM-14-0-s001.docx]

**Supporting Information**

**Biomimetic Co-delivery of Lenvatinib and FePt Nanoparticles for Enhanced Ferroptosis/Apoptosis Treatment of Hepatocellular Carcinoma**

*Feichao Xuan, Xingyang Zhao, Weiran Pang, Zirong Li, Xiangyi Yin, Weizhong Xie, Xiaojun Zeng,* *Liming Nie, Junying Yang, shiying Li, Puxiang Lai^*^, Chihua Fang*^*^

**
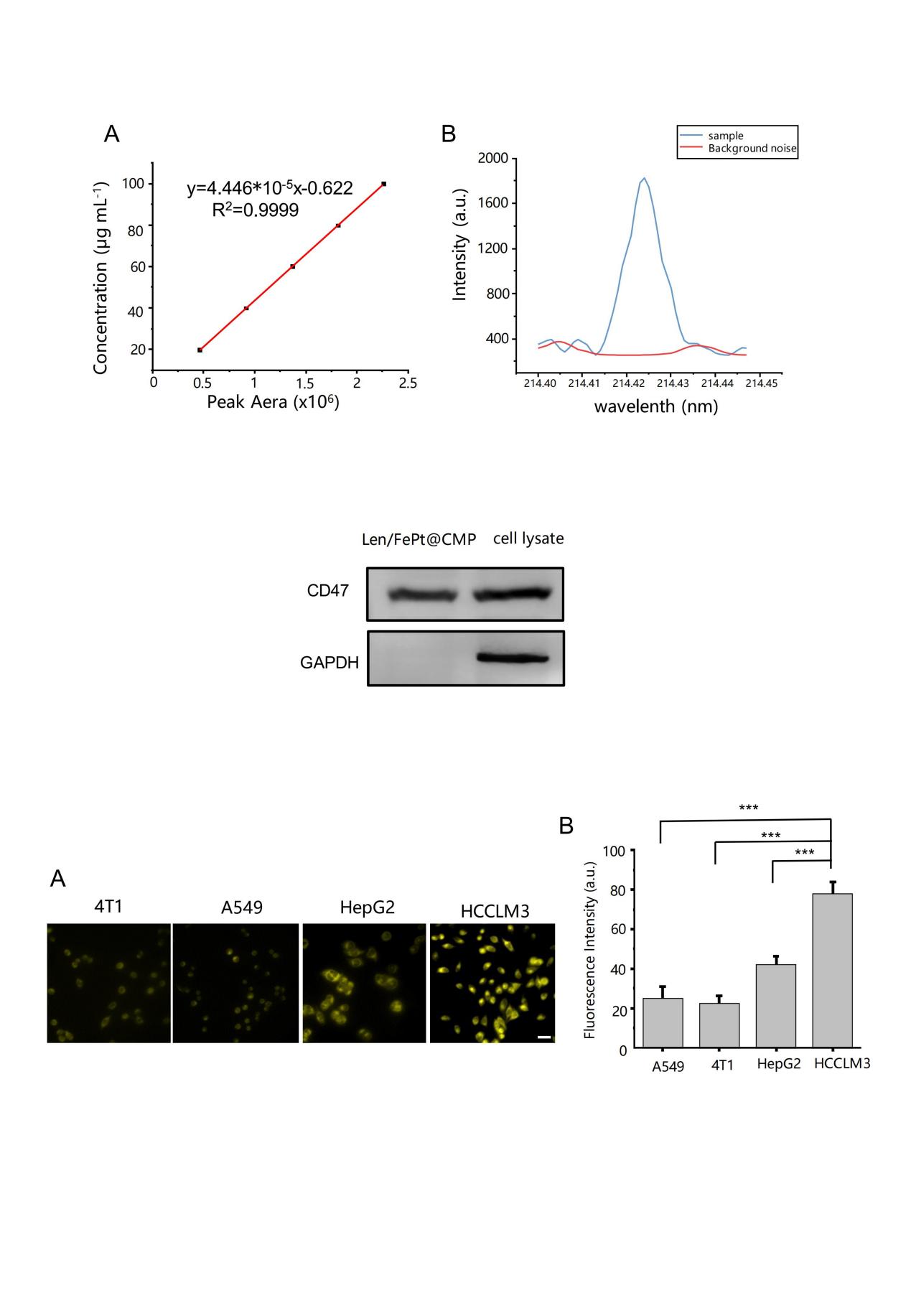
**

**Figure S1. (A)** Standard curves of Len concentration using HPLC. **(B)** Measurement curves of platinum concentration using ICP-OES.


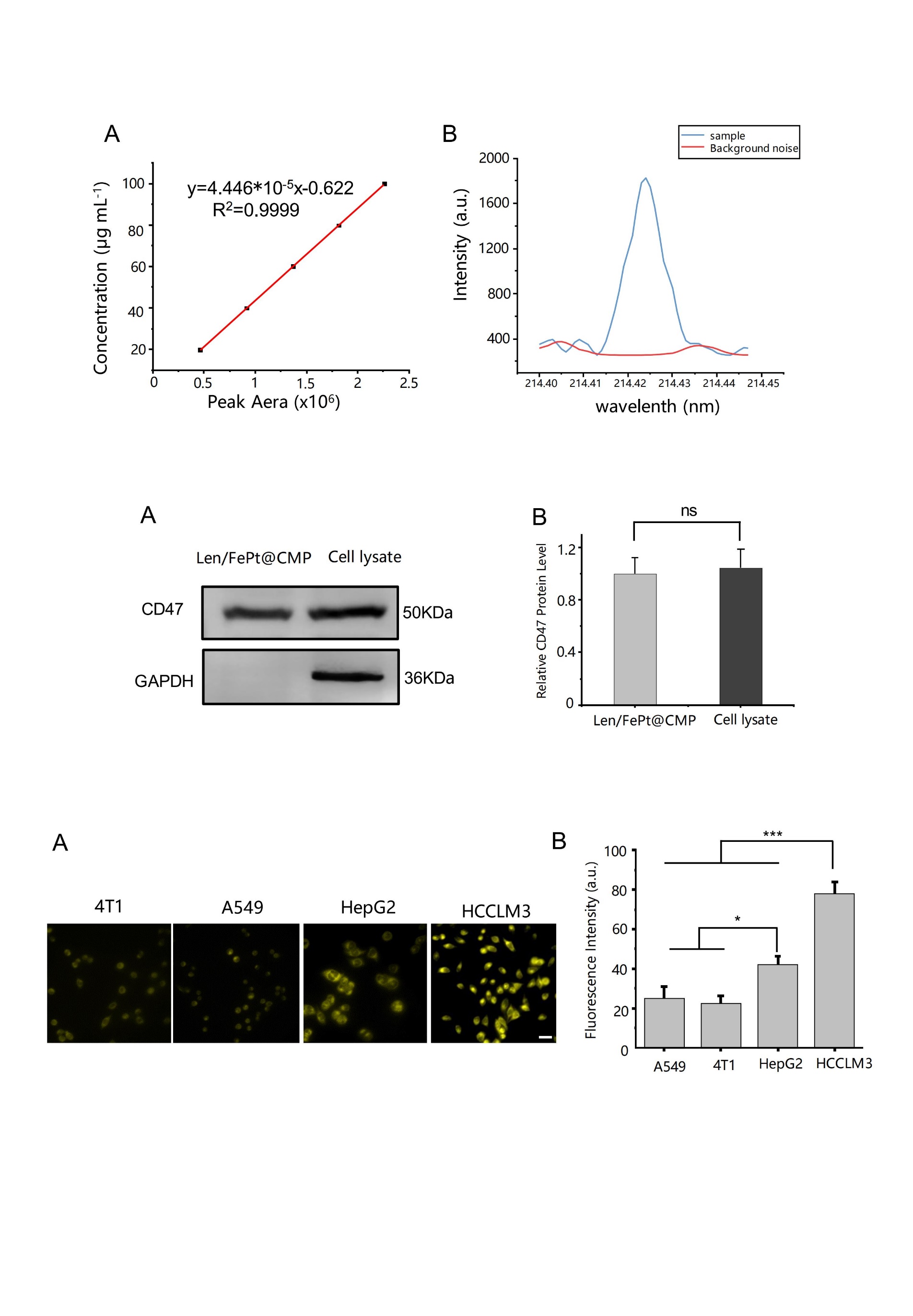


**Figure S2. (A)** Western blot analysis of CD47 on Len/FePt@CMP NPs and HCCLM3 cell lysate. **(B)** Quantitative measurement of CD47 protein in A.


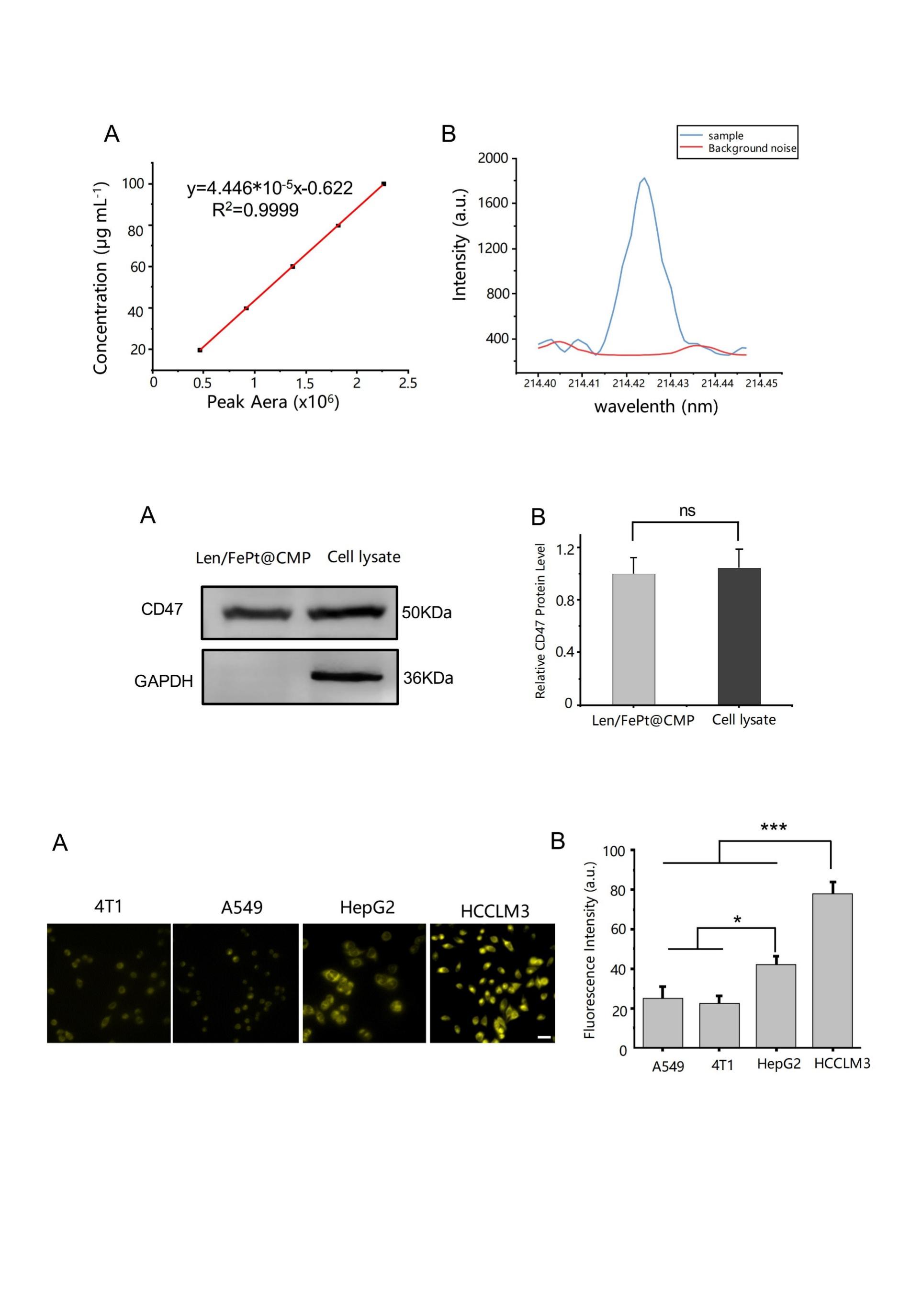


**Figure S3.** **(A)** Fe^2+^ fluorescence images of HCCLM3, A549, HepG2 and 4T1 cells treated with Len/FePt@CMP NPs (Scale bar: 50 µm). **(B)** Quantification of fluorescence intensity in A (*P value< 0.05, ***P value< 0.001).


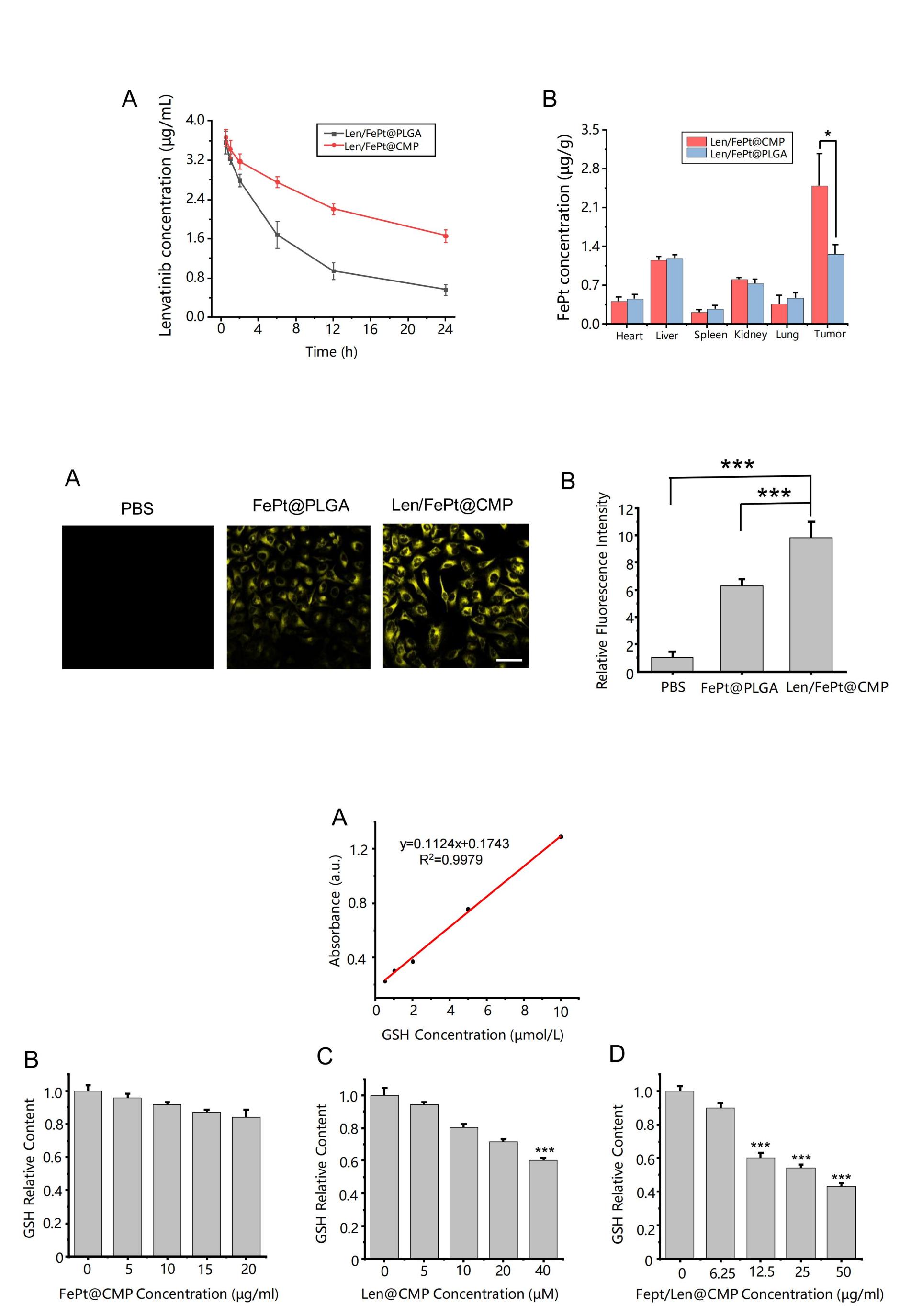


**Figure S4. (A)** Fe^2+^ fluorescence images of HCCLM3 cells treated with PBS, Len/FePt@PLGA and Len/FePt@CMP NPs (Scale bar: 50 µm). **(B)** Quantification of Fe^2+^ fluorescence intensity in A (***P value< 0.001).


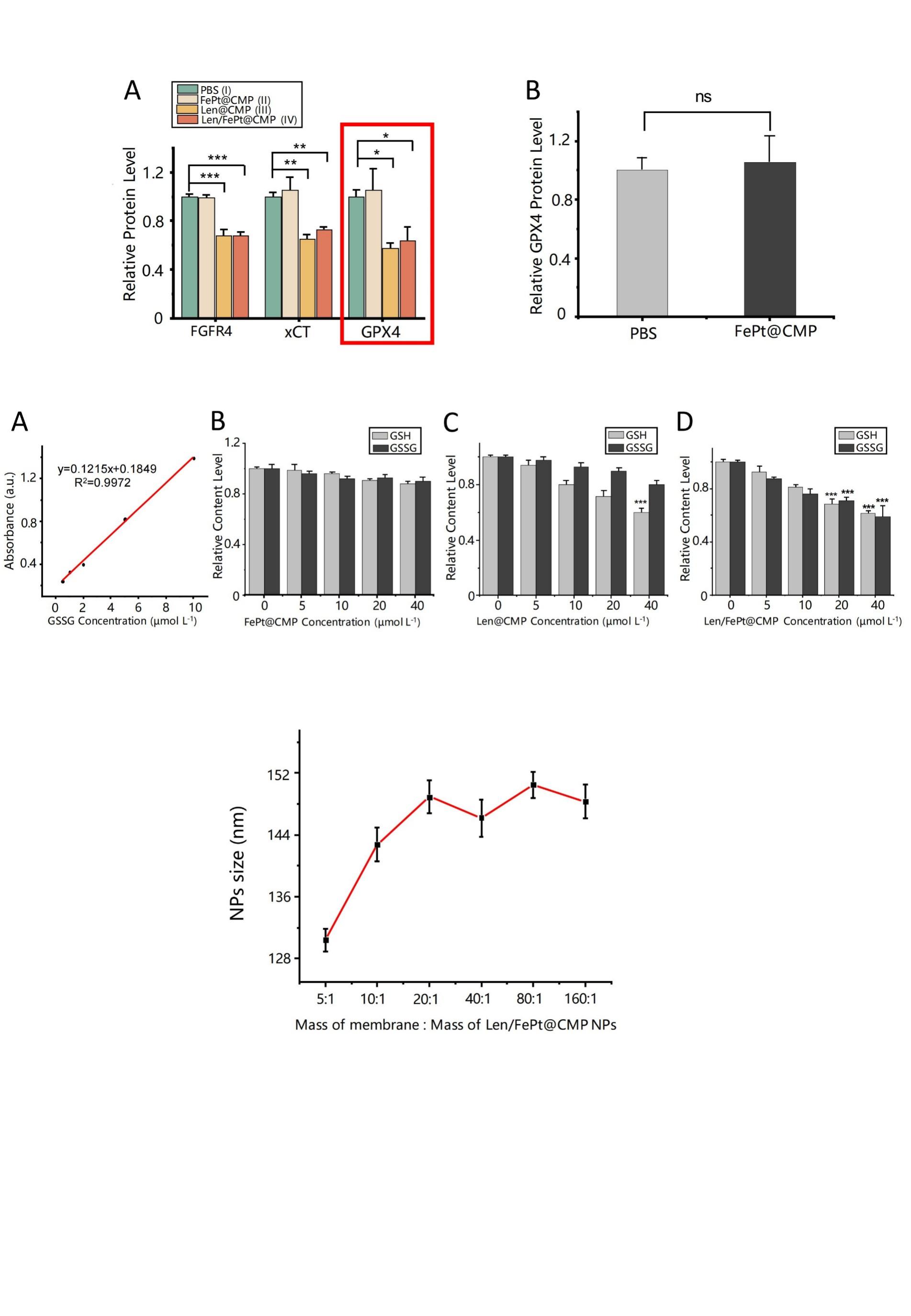


**Figure S5. (A)** Standard curve of the Glutathione detection kit. **(B)** GSH and GSSG level detection of HCCLMC cells treated with FePt@CMP NPs. **(C)** GSH and GSSG level detection of HCCLMC cells treated with Len@CMP NPs (***P value< 0.001). **(D)** GSH and GSSG level detection of HCCLMC cells treated with Len/FePt@CMP NPs (***P value< 0.001).


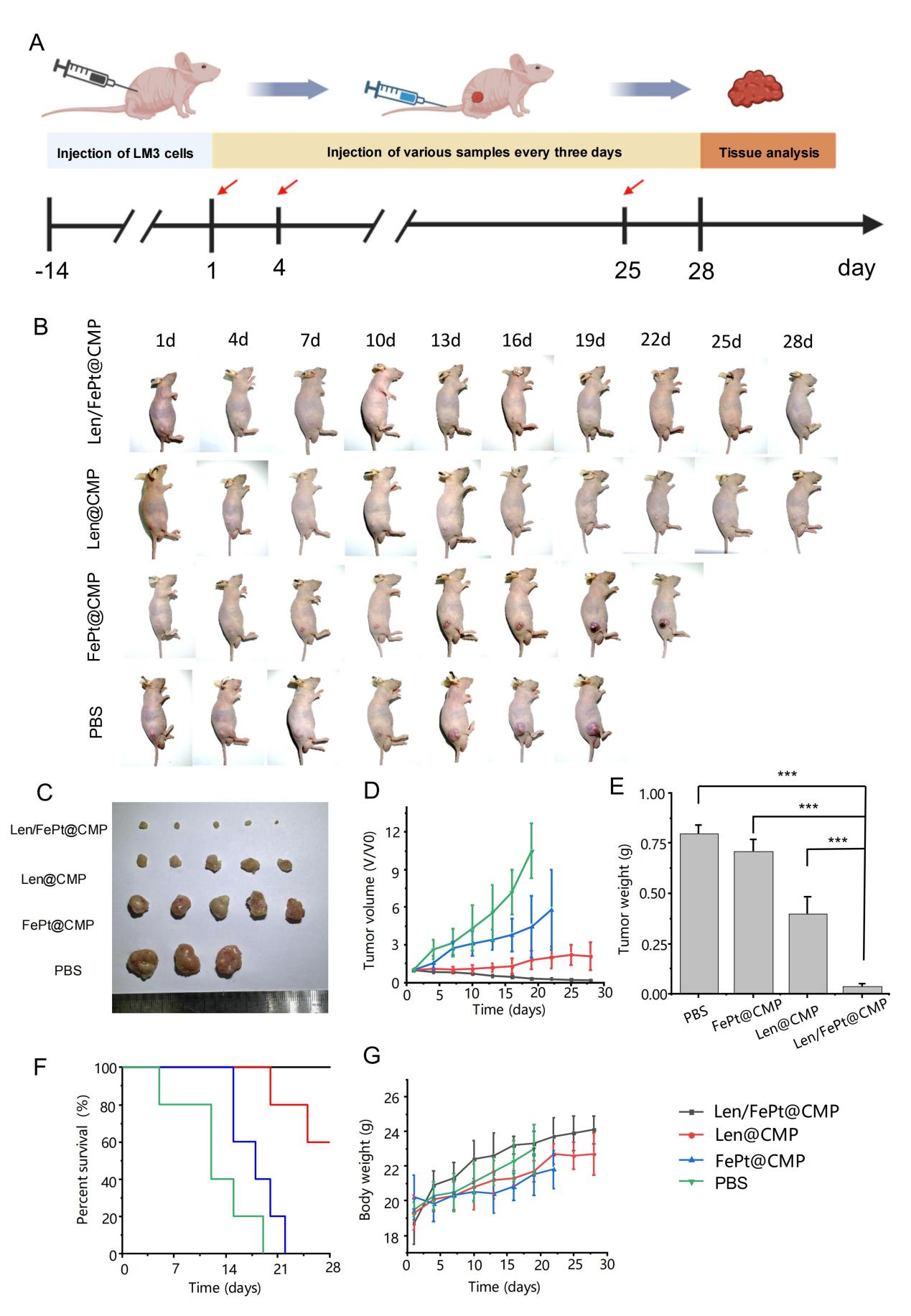


**Figure S6. (A)** Illustration of the treatment procedure. **(B)** Representative photographic images of tumor bearing mice under surveillance in each treatment group (n=5). **(C)** Photographic images of tumors harvested from mice after termination of the monitoring period. **(D)** Relative tumor volume curves. **(E)**Tumor weights of mice in the various groups (***P value< 0.001). **(F)** Survival in each group during treatment period. **(G)** The body weight changes of mice observed in each treatment group.


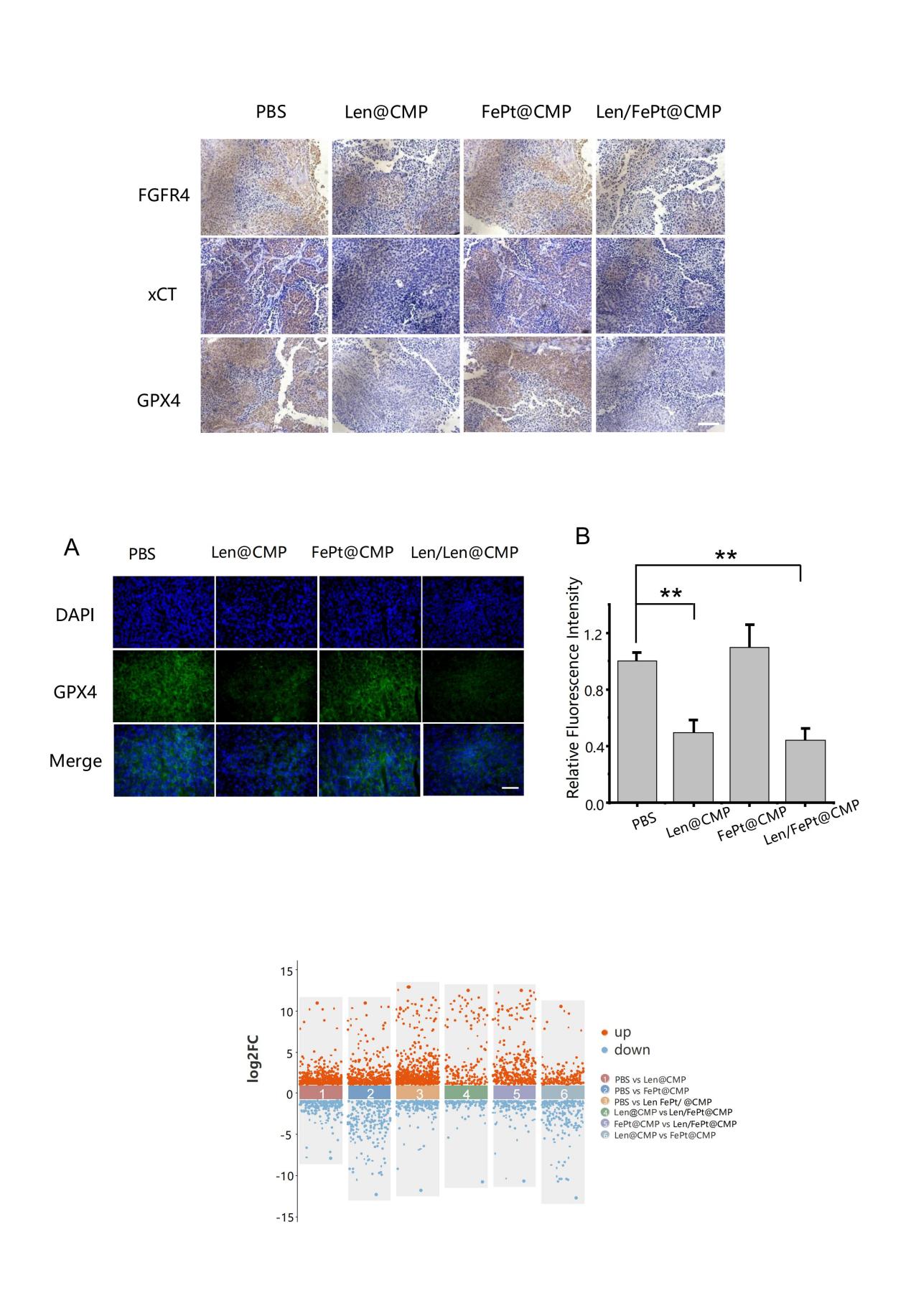


**Figure S7. (A)** GPX4 staining of tumor tissue in each treatment group (scale bar: 50 µm). **(B)** Quantification of fluorescence intensity analysis in A (**P value< 0.01).


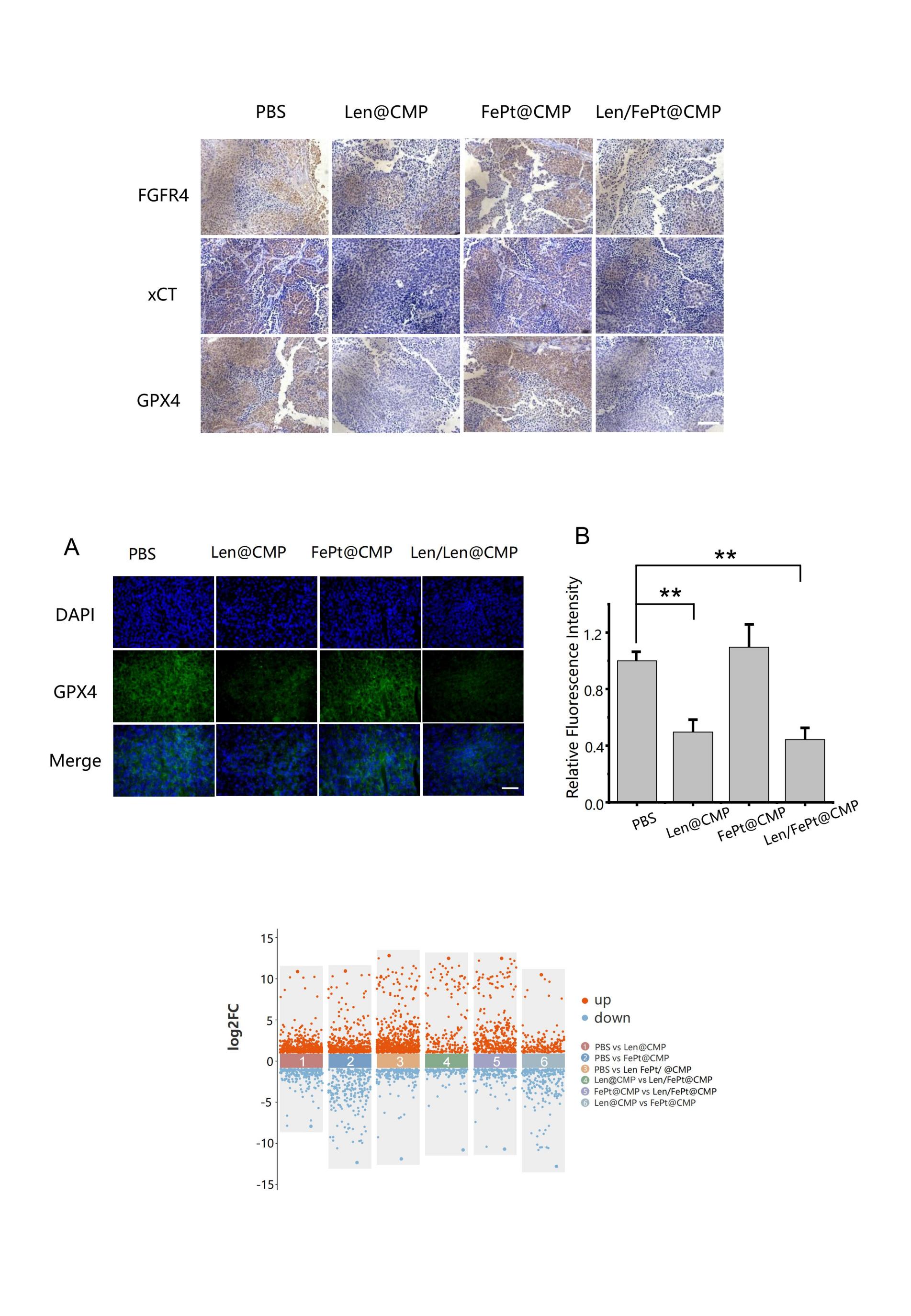


**Figure S8.** Immunohistochemical staining of tumor tissue showing the changes in the expression of FGFR4, xCT and GPX4 (scale bar: 100 µm).


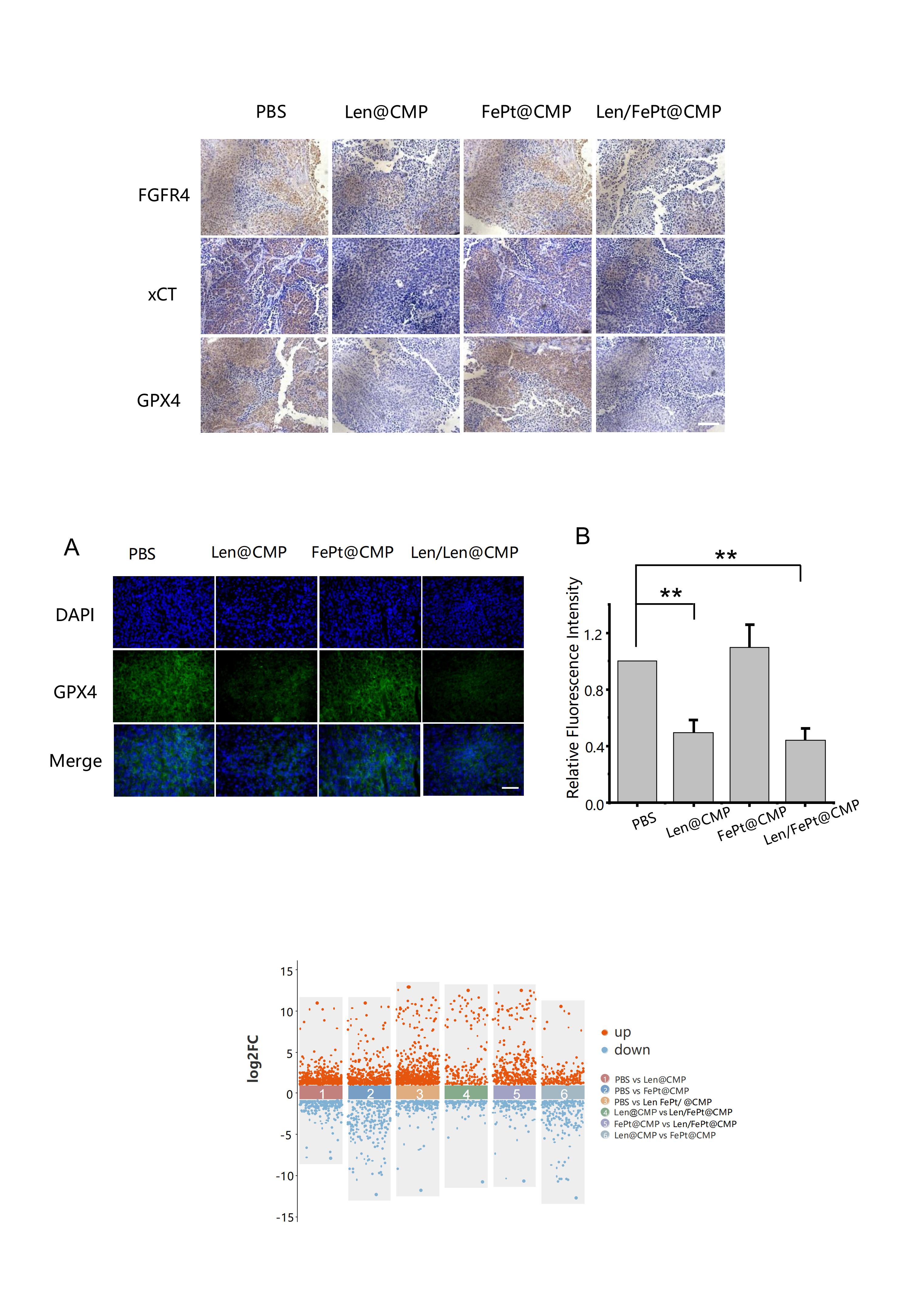


**Figure S9.** The distribution of the differentially expressed genes (DEGs) between different groups.


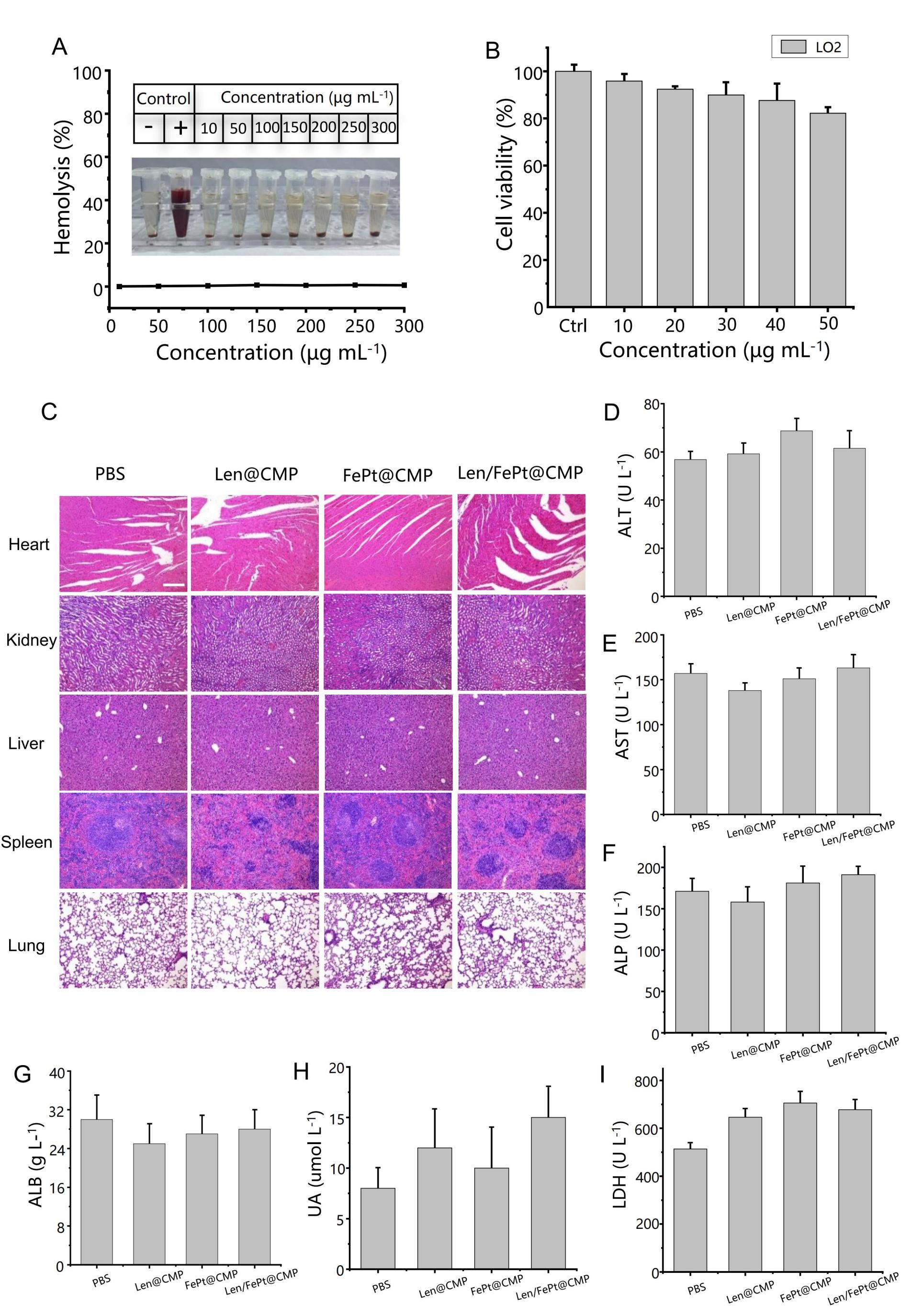


**Figure S10. (A)** Hemolysis assays of Len/FePt@CMP NPs. **(B)** Cytotoxicity of Len/FePt@CMP NPs after the incubation with LO2. **(C)** HE staining of main organs (heart, liver, spleen, lung and kidney) collected from mice after various treatment. (scale bar: 250 um). Serum biochemical indexes including ALT **(D)**, AST **(E)**, ALP **(F)**, ALB **(G)**, UA **(H)** and LDH **(I)** of the mice after various treatment (ALT: alanine transaminase, AST: glutamic oxaloacetic transaminase, ALP: alkaline phosphatase, ALB: albumin, UA: uric acid, LDH: lactate dehydrogenase).
